# Dietary intake and the risk of monoclonal gammopathy of undetermined significance: results from the population-based iStopMM screening study

**DOI:** 10.1038/s41408-026-01480-4

**Published:** 2026-04-08

**Authors:** Styrmir Hallsson, Ingibjorg Gunnarsdottir, Marianna Thordardottir, Sigrun Thorsteinsdottir, Johanna E. Torfadottir, Isleifur Olafsson, Ingunn Thorsteinsdottir, Jon Thorir Oskarsson, Andri Olafsson, Thorvardur J. Love, Sigurður Yngvi Kristinsson, Sæmundur Rögnvaldsson

**Affiliations:** 1 Faculty of Food Science and Nutrition, University of Iceland, Reykjavík, Iceland; 2 Landspítali – The National University Hospital of Iceland, Reykjavík, Iceland; 3 Faculty of Medicine, University of Iceland, Reykjavík, Iceland; 4 Department of Hematology, Rigshospitalet, Copenhagen, Denmark; 5 Centre for Public Health Sciences, University of Iceland, Reykjavik, Iceland; 6 Department of Hematology and Coagulation, Sahlgrenska University Hospital, Gothenburg, Sweden; 7 Institute of Meidince, Sahlgrenska Academy, University of Gothenburg, Gothenburg, Sweden

**Keywords:** Risk factors, Myeloma

## Abstract

Monoclonal gammopathy of undetermined significance (MGUS) is the asymptomatic precursor of multiple myeloma (MM) and related disorders. Understanding the causes of MGUS is key to elucidating the causes of these malignancies. The association of MGUS and diet, including dietary patterns, remains unclear. We therefore assessed the relationship between diet and MGUS in the iStopMM a nationwide screening study for MGUS in Iceland where 75,422 individuals have been screened for MGUS. A food-frequency questionnaire was distributed to participants with 27,217 respondents, of whom 1,020 had MGUS at screening. Exposure was defined as dietary intake of specific dietary items and adherence to five dietary patterns identified through principal component analysis. Logistic regression was used to calculate odds ratios (OR) of having MGUS, adjusting for age, sex, physical activity, and education. No dietary patterns nor specific dietary components were associated with risk of MGUS overall. However, high consumption of dairy products was associated with increased odds (OR 2.01, 95% CI 1.16-3.65) of IgA MGUS, even after adjusting for multiple comparisons. These findings suggest that dietary habits are not a major risk factor for MGUS overall and therefore unlikely to be a major causative factor in the development of MM.

## Introduction

Monoclonal gammopathy of undetermined significance (MGUS) is a precursor to multiple myeloma (MM) and related hematologic malignancies [1]. MGUS is an asymptomatic condition characterized by the presence of monoclonal immunoglobulins (M proteins) or free light chains (FLCs) in the serum [2] with a prevalence of 4–5% after 40 years old [3]. The main clinical implication of MGUS is a 0.5–1.5% risk of progression to MM or related lymphoproliferative disorder per year [4]. MGUS can be categorized by the M protein heavy chain isotype into IgG, IgA, and IgM MGUS and by the absence of M proteins in the presence of an abnormal FLC ratio as light-chain MGUS [4].

The etiology of MGUS and its progression to active disease remains poorly understood; however, several risk factors have been identified [5]. The prevalence of MGUS increases with age and is higher in males [5]. Other known risk factors include ethnicity, with higher prevalence in African Americans [6], a familial history of lymphoproliferative disorders, including MM and MGUS [7], and exposure to pesticides [8]. Obesity has been associated with increased risk of MGUS and MM [9–11]. Certain dietary habits have been associated with MM risk, with some studies suggesting a protective effect of higher intake of fish and vegetables [12–14]. Vegetarian and vegan diets have also been associated with reduced risk of MM [15, 16]. Conversely, a higher score in the “Empirical dietary inflammatory pattern”, has been associated with increased MM risk [17]. A diet characterized by high intake of fruits and vegetables, whole grains, nuts, and legumes, the “prudent” dietary pattern, has been linked to lower MM-specific mortality, while the “Western” dietary pattern, with higher intake of processed and red meats, sugars, and a lower intake of fruits and vegetables, has been associated with increased MM-specific mortality [18]. Overall, these studies suggest that higher intake of fruits and vegetables may confer a protective effect, while diets high in processed meats and sugars may increase MM risk. However, these studies are mostly small, retrospective, and use varying dietary assessment methods, leading to inconsistent findings.

Since all cases of MM are preceded by MGUS, it is of particular interest whether the same dietary risk factors apply to MGUS. A study from the NHANES cohort in the US has observed increased risk of MGUS in higher consumption of soft drinks and red meat. Conversely, a lower risk was seen for higher consumption of fruits and vegetables and wholegrain products [19]. A study using data from the German Heinz-Nixdorf study cohort showed a lower risk solely for higher fruit intake in women [20]. Similarly, results from the Icelandic AGES study cohort showed a decreased risk with higher adolescent fruit consumption and higher midlife consumption of whole wheat bread [21]. However, most previous studies have included relatively small numbers of MGUS cases. Furthermore, these prior studies have not assessed the dietary risk factors for different MGUS isotypes, even though they have different pathobiology and patterns of progression. In particular, IgM and non-IgM MGUS subtypes which are caused by the presence of clonal lymphoplasmacytic lymphocytes or mature B lymphocytes and clonal plasma cells, respectively [22]. Furthermore, IgA MGUS has been associated with a higher risk of progression [5] and is of particular interest with regard to dietary intake since IgA is normally produced in response to immune stimuli in the gut [23]. Given this heterogeneity between MGUS isotypes, it is of special interest to examine the dietary risk factors of these different MGUS subtypes.

In this study, we used data from the Iceland Screens, Treats, or Prevents Multiple Myeloma study (iStopMM), a population-based screening study for MGUS in Iceland, where 75,422 have been screened for MGUS. The aim of this study was to assess the relationship between dietary intake as individual dietary components and as dietary patterns, and MGUS and its isotypes.

## Methods

### Ethical considerations

The study protocol, all information material, biobank, and questionnaires were approved by the Icelandic National Bioethics Committee (Number 16–022, date: 2016-04-26) with approval from the Icelandic Data Protection Agency. Access to national healthcare registries was approved by the Icelandic Directorate of Health and the Icelandic Cancer Society. All participants of the iStopMM study provided written informed consent, including for this study. All methods were performed in accordance with the relevant guidelines and regulations.

### Study cohort

Study data was acquired from the iStopMM study, a nationwide population-based, prospective screening study and randomized trial of follow-up strategies. In total, 75,422 participants (~50% of the total Icelandic population over 40 years old) were screened for MGUS using serum protein electrophoresis (SPEP) and FLC assay between 2016 and 2020. M-protein concentration, MGUS isotype, and FLC ratio were also obtained from the screening samples, with diagnosed MGUS samples being further classified into MGUS with or without M proteins (heavy-chain and light-chain MGUS) and into M protein isotypes. Participants with MGUS were subsequently randomized to three study arms to investigate the role of follow-up for MGUS and evaluate the potential benefits and harms of screening with two thirds with MGUS being notified and entering active follow-up [24].

### Dietary intake

A validated food frequency questionnaire (FFQ) [25] was sent out in the spring of 2023 and was used to gather information about the diet of iStopMM study participants. In total, 27*,*217 answered the FFQ, of whom 1020 had MGUS at screening (3.75%), of which two thirds knew of their MGUS diagnosis when answering the FFQ. The study design is therefore a retrospective case control study.

The FFQ included a total of 123 questions, 15 questions about socioeconomic background, 8 questions about physical activity, and 100 questions about dietary intake for the last 12 months and 1 question assessing adolescent intake of dairy products. The FFQ included the following response variables for frequency of intake of food from different food groups: *“Seldom/never”, “1-3 times a month”, “1-2 times a week”, “3-4 times a week”, “5-6 times a week”*, and *“Daily/almost daily”*.

Questions about main meal types such as fish, meat, pasta, pizza, poultry, and vegetarian meals had an additional answering alternative with “*1 time a week”* and *“2 times a week”*. Answers from questions regarding consumption of food from selected food groups (red meat, poultry, fish, dairy, fruits, vegetables, and wholegrain bread products) were converted to numerical values, summed, and reported as frequency of consumption per week.

### Extraction of dietary patterns

We used Principal Component Analysis (PCA) to extract discrete dietary patterns. PCA is a data driven technique used to reduce the complexity and dimensionality of data [26]. In short, the method was used to detect dietary patterns with key dietary elements represented by principal component loadings. Component loadings for dietary variables that were higher than 0.30 or lower than −0.30 were used to define the dietary patterns. Components with eigenvalues > 1 as well as the results from a parallel analysis (PA) were used to decide on the number of dietary patterns to retain [26] (see Supplementary Table 4 and Supplementary Fig. 1). Individual adherence to the specific dietary patterns can be calculated based upon their FFQ answers, giving them scores. An orthogonal varimax PCA was used to create clear component loadings and thus more distinct dietary patterns, which better represent the diet of the population [27]. Questions covering the different food types and frequency of their consumption, a total of 67 questions from the FFQ were used for this analysis. The answers from the FFQ were turned into numerical values, which were then standardized by centering and scaling the data. The standardization allowed for creating a correlation matrix from the food variables with different answering alternatives affecting the data equally [26]. Bartlett´s test of sphericity was used to assess whether the data was suitable for PCA by evaluating the intercorrelation between the variables analyzed, deeming the data fit. In addition, a Kaiser-Meyer-Olkin test (KMO test) was performed to measure adequate sampling of the data [28]. The overall measure of sampling adequacy (MSA) was 0.85 with all individual MSA values of variables included being higher than 0.60. This shows that the correlation between the variables was primarily due to shared variance across all variables, making the dataset excellent for performing PCA. Food variables with correlation coefficients over 0.30 were used to identify the dietary patterns, as these contribute substantially to the extracted principal component. Based on the adherence to the identified dietary patterns, a dietary pattern score was calculated and used to place individuals into quartile groups, demonstrating high to low adherence to different dietary patterns. A PCA was performed on both the complete case- and the imputed FFQ data.

### Missing data

In order to account for missing data, we used imputed unanswered questions in cases where participants left blank answers. Sampling studies have demonstrated that bias is reduced when comparing imputed data to a complete case analysis [29]. Single imputation (SI) using predictive mean matching (PMM) was used to fill in for missing values in the FFQ. As the data needed to be prepared further before conducting logistic regression a single imputation was performed. The MICE function was used to impute the missing values. In total, 20 iterations were performed in the imputation, achieving convergence for the imputed values.

In total, 25,307 participants (93% of total participants, 91% of MGUS cases) had an answer rate equal to or greater than 90% for the FFQ-questions. Additionally, a complete case analysis was performed removing individuals with missing data in any of the 67 FFQ questions included in the PCA. No difference in dietary patterns nor the dietary components was observed between the two approaches.

### Statistical analysis

All main statistical analyses were based on the imputed data set, reducing bias that can occur from missing values [29, 30]. Logistic regression analysis was used to evaluate the odds ratio (OR) of having MGUS based on the different dietary patterns. The lowest quartile was used as a reference group for the analysis. All models were adjusted for age and sex. Additional models were also performed, adjusting for age, sex, education level, and physical activity. An additional sensitivity analysis was performed, including BMI, which was available for a subset of the participants. Additional analysis was performed for individuals unknowing of their MGUS diagnosis (Supplementary Table 9). Complete case analysis (CCA) was also performed for all dietary patterns and components (Supplementary Tables 6–8).

In addition to the main analysis, subgroup analyses were performed. Logistic regression evaluating the OR of having different MGUS isotypes based on diet was performed. The same groups for dietary component consumption and dietary patterns were used in these analyses. Intra-isotype analyses, including only participants diagnosed with MGUS, as well as analyses including all participants in the FFQ-cohort, were performed. These analyses assessed the dietary effects on M protein isotypes, looking at IgA, IgM, and IgG individually.

Mann-Whitney U test was performed for non-parametric data (age and BMI) to evaluate statistical significance of differences observed between groups. A Chi-square test was performed to see if there was a significant difference between the sex proportion of the groups as well as the education status and physical level.

All statistical analyses were performed in R Statistical Software (v.4.2.2; R Core Team 2022) [31]. The package “mice” [32] was used to perform the imputation of missing values and the packages “FactoMineR” [33], “Factoextra” [34], “corrr” [35], “nFactors” [36], “GPArotation” [37] for the PCA.

## Results

Of the 75,422 participants screened 27,217 answered the FFQ, of whom 1020 had MGUS at screening (3.75%). Due to the iStopMM study design, approximately 2/3 of participants with MGUS knew about their diagnosis. The distribution of MGUS isotypes in those who answered the FFQ-cohort was similar to the whole iStopMM cohort. Participants who answered the FFQ questionnaire were significantly older than that the overall iStopMM cohort (mean age 63 years vs. 59 years, *p* < 0.001) (Supplementary Table 1). Median time between screening and answering the FFQ was 5 years (range: 3–7 years). The proportion of males was significantly higher in the MGUS group (55.9% in MGUS group vs. 49% in non-MGUS group, *p* < 0.001) (Table 1).

**Table 1:** Background information of participants who gave a response to the food frequency questionnaire - MGUS vs. Non-MGUS (Non-imputed).

	MGUS	Non-MGUS
*n* (%)	1020 (3.75%)	26197
Median age (IQR)**	65 (57–71)	59 (51–66)
Age range	41–88	41–92
*n* Males	549	11711
*n* Females	471	1448
% male**	53.8%	44.7%
Median BMI—kg/m^2^ (IQR)	27.62 (25.01–30.25)	27.73 (25.00–30.93)
Education level (%)**		
Dropped out	−23 (2.3%)	−462 (1.8%)
Primary school	−203 (20.4%)	−4408 (16.8%)
Secondary school	−199 (20.0%)	−5303 (20.2%)
Technical school	−220 (22.1%)	−5090 (19.4%)
University degree	−321 (32.3%)	−10423 (39.7%)
Missing	−28 (2.8%)	−537 (2.0%)
Physical activity		
Never	−29 (2.9%)	−938 (3.5%)
<1 per week	−121 (12.1%)	−3071 (11.7%)
1–2 per week	−164 (16.5%)	−5140 (19.6%)
3–4 per week	−303 (30.5%)	−8011 (30.5%)
5–6 per week	−206 (20.7%)	−4821 (18.4%)
Daily	−170 (17.1%)	−4135 (15.7%)
Missing	−1 (0.1%)	−107 (0.4%)
*n* fully completed FFQ (%)^a^	494 (48.4%)	14612 (55.8%)
*n* ≥ 90% completed FFQ (%)	928 (91.0%)	24379 (93.1%)

*IQR* interquartile range.

^a^Individuals who answered all 67 FFQ-questions

**p-value < 0.01.

### Dietary patterns

Five, distinct dietary patterns were identified: the “*fruit & vegetable pattern*”, *“red meat pattern”, “sweet tooth pattern”, “bread pattern*”, and the *“fish meal pattern”* (Supplementary Table 3). Eigenvalues of all principal components (dietary patterns) were >1.5 with the lowest eigenvalue being 1.71 and the highest 4.81 (Supplementary Table 4). No variables contributed highly in a negative way to the dietary patterns (no correlation coefficients were below −0.30), (Supplementary Figs. 2–6).

No significant association was observed between adherence to a specific dietary pattern and MGUS overall (Table 2). The crude models showed significant OR´s for the *“sweet tooth pattern”* and the *“bread pattern”* but the effect disappeared after the adjustment of covariates.

**Table 2:** Associations between the different dietary patterns and MGUS.

Dietary pattern		Quartile 1 (*n* = 6805)	Quartile 2 (*n* = 6804)	Quartile 3 (*n* = 6804)	Quartile 4 (*n* = 6804)
*n* = 27217		OR (95% CI)	OR (95%CI)	OR (95%CI)	OR (95% CI)
Fruit and vegetable pattern	Crude	Ref.	0.89 (0.74–1.06)	0.84 (0.70–1.01)	0.94 (0.79–1.12)
	Adjusted for age and sex	Ref.	0.90 (0.76–1.08)	0.87 (0.73–1.05)	0.94 (0.78–1.12)
	Adjusted for age, sex, education, and physical activity	Ref.	0.90 (0.76–1.08)	0.88 (0.73–1.05)	0.94 (0.78–1.13)
Red meat pattern	Crude	Ref.	0.98 (0.82–1.17)	0.97 (0.81–1.16)	0.94 (0.78–1.12)
	Adjusted for age and sex	Ref.	0.97 (0.81–1.16)	0.98 (0.82–1.17)	0.97 (0.81–1.16)
	Adjusted for age, sex, education, & physical activity	Ref.	0.96 (0.81–1.15)	0.97 (0.81–1.17)	0.96 (0.80–1.15)
Sweet tooth pattern	Crude	Ref.	0.94 (0.80–1.11)	0.77 (0.64–0.91)	0.62 (0.5–0.75)
	Adjusted for age and sex	Ref.	1.13 (0.95–1.33)	1.00 (0.84–1.20)	0.98 (0.81–1.18)
	Adjusted for age, sex, education, and physical activity	Ref.	1.13 (0.95–1.33)	1.00 (0.84–1.20)	0.98 (0.81–1.19)
Bread pattern	Crude	Ref.	1.24 (1.03–1.50)	1.21 (0.99–1.46)	1.49(1.24–1.79)
	Adjusted for age and sex	Ref.	1.14 (0.95–1.38)	0.96 (0.80–1.17)	1.00 (0.83–1.21)
	Adjusted for age, sex, education, and physical activity	Ref.	1.14 (0.95–1.38)	0.96 (0.80–1.16)	1.00 (0.83–1.20)
Fish meal pattern	Crude	Ref.	0.83 (0.69–0.99)	0.88 (0.74–1.05)	0.95 (0.80–1.13)
	Adjusted for age and sex	Ref.	0.82 (0.68–1.00)	0.86(0.71–1.03)	0.92 (0.76–1.10)
	Adjusted for age, sex, education, and physical activity	Ref.	0.83 (0.69–1.00)	0.88 (0.73–1.06)	0.94 (0.79–1.13)

*OR* odds ratio, 95% *CI* 95% confidence interval, *Ref* reference group.

### Dietary components

Neither consumption of the analyzed dietary components, nor being in a higher percentile group showed any significant effect on the odds of having MGUS in the models adjusted for age and sex, and age, sex, education, and physical activity. Specific dietary components were not found to be associated with MGUS risk overall (Table 3).

**Table 3:** Associations between dietary components and MGUS.

Dietary component		Quartile 1	Quartile 2	Quartile 3	Quartile 4
*n* = 27217		OR (95% CI)	OR (95%CI)	OR (95%CI)	OR (95% CI)
Meat products	Crude	Ref.	0.75 (0.61–0.93)	0.81 (0.68–0.96)	0.80 (0.69–0.94)
	Adjusted for age and sex	Ref.	0.83 (0.67–1.03)	0.95 (0.80–1.13)	1.00 (0.85–1.17)
	Adjusted for age, sex, education, and physical activity	Ref.	0.83 (0.67–1.03)	0.95 (0.80–1.13)	1.00 (0.85–1.17)
Red meat	Crude	Ref.	1.08 (0.92–1.26)	1.00 (0.78–1.26)	1.07 (0.90–1.26)
	Adjusted for age and sex	Ref.	1.07 (0.91–1.25)	0.98 (0.76–1.24)	1.04 0.88–1.24)
	Adjusted for age, sex, education, and physical activity	Ref.	1.06 (0.91–1.25)	0.98 (0.76–1.24)	1.04 (0.87–1.25)
Poultry^a^	Crude	Ref.	0.76 (0.65–0.88)		0.61 (0.53–0.71)
	Adjusted for age and sex	Ref.	0.92 (0.79–1.07)		0.90 (0.77–1.05)
	Adjusted for age, sex, education, and physical activity	Ref.	0.92 (0.78–1.07)		0.90 (0.77–1.05)
Fish	Crude	Ref.	0.93 (0.78–1.11)	1.41 (1.14–1.73)	1.40 (1.18–1.66)
	Adjusted for age and sex	Ref.	0.84 (0.71–1.01)	1.10 (0.89–1.35)	0.95 (0.80–1.13)
	Adjusted for age, sex, education, and physical activity	Ref.	0.84 (0.70–1.01)	1.09 (0.88–1.34)	0.95 (0.80–1.13)
Dairy	Crude	Ref.	1.28 (1.06–1.54)	1.39 (1.15–1.68)	1.61 (1.34–1.93)
	Adjusted for age and sex	Ref.	1.25 (1.03–1.51)	1.13 (0.93–1.37)	1.11 (0.92–1.34)
	Adjusted for age, sex, education, and physical activity	Ref.	1.24 (1.03–1.50)	1.13 (0.93–1.37)	1.10 (0.91–1.33)
Fruits^a^	Crude	Ref.	1.09 (0.92–1.29)		1.16 (1.00–1.33)
	Adjusted for age and sex	Ref.	1.06 (0.89–1.26)		0.99 (0.86–1.15)
	Adjusted for age, sex, education, and physical activity	Ref.	1.06 (0.89–1.26)		1.00 (0.86–1.15)
Vegetables	Crude	Ref.	0.96 (0.80–1.14)	0.86 (0.72–1.03)	0.86 (0.73–1.01)
	Adjusted for age and sex	Ref.	1.00 (0.84–1.20)	0.93 (0.78–1.12)	0.95 (0.81–1.12)
	Adjusted for age, sex, education, and physical activity	Ref.	1.00 (0.84–1.21)	0.93 (0.77–1.12)	0.96 (0.81–1.13)
Wholegrain bread^a^	Crude	Ref.	1.13 (0.95–1.34)		1.48 (1.29–1.71)
	Adjusted for age and sex	Ref.	1.02 (0.85–1.21)		1.00 (0.86–1.16)
	Adjusted for age, sex, education, and physical activity	Ref.	1.01 (0.85–1.21)		1.00 (0.86–1.16)

^a^Due to low variance in distribution of fruit, poultry, and wholegrain consumption individuals were grouped into tertiles instead of quartiles for these analyses.

### MGUS isotype analysis

Dietary pattern adherence and consumption of individual dietary components were not associated with the different MGUS isotypes, except for an association seen between IgA MGUS and dairy consumption (Table 4). The top consumption group (>10 times per week) had twice the odds of having IgA MGUS (OR = 2.01; 95% confidence interval (CI): 1.16–3.65; *p* = 0.016) compared to the reference group (<1.5 times per week) (Table 4). While only the top consumption group was significantly different from the reference group there was a clear dose effect for higher consumption of dairy.

**Table 4:** Association between IgA MGUS and dairy consumption.

Dairy	Quartile 1	Quartile 2	Quartile 3	Quartile 4
*n* IgA MGUS: 123	OR (95% CI)	OR (95%CI)	OR (95%CI)	OR (95% CI)
Crude	Ref.	1.51 (0.83–2.84)	2.11 (1.19–3.87)	2.74 (1.60–4.90)*
Adjusted for age and sex	Ref.	1.48 (0.81–2.78)	1.78 (1.00-3.28)	2.01 (1.16–3.65)**
Adjusted for age, sex, education, and physical activity	Ref.	1.47 (0.80–2.77)	1.77 (1.00–3.27)	1.97 (1.14–3.57)***

*OR* odds ratio, 95% *CI* 95% confidence interval.

*P-value: <0.01 **P-value: 0.016. ***P-value: 0.02.

### Sensitivity analysis

Several sensitivity analyses were performed in addition to the main analysis performed on the imputed data for dietary components as well as the dietary patterns for all MGUS and the different MGUS isotypes. 15,106 individuals (494 MGUS cases) were used in the complete case analysis (CCA), where no difference in the results was observed compared to the main analysis (imputed data). The complete case analysis for dairy consumption and IgA MGUS showed similar results, with the highest quartile having OR 2.95 (95% CI 1.30–7.38). A multivariate logistic regression adjusting for age, sex, education, weekly physical activity as well as BMI did not show any significant results. Too few BMI values were available to assess IgA MGUS with dietary intake. Additional analysis, including only individuals that knew of their MGUS diagnosis, and the controls did not result in any significant associations for neither dietary components nor dietary patterns. In addition, to compare the dietary scores between the study arms (arms 1–3) an ANOVA was performed, resulting in no significant differences between the groups. Finally, a sensitivity analysis investigating a possible risk reduction between daily wholegrain consumption, daily fruit consumption, daily vegetable consumption and MGUS was performed, where no association was observed. The results are available in the supplemental data (Supplementary Table 10).

## Discussion

In this largest study to date on the association of dietary intake and risk of MGUS based on population-based study including 27,200 individuals screened for MGUS, we found no meaningful association between the dietary patterns analyzed, specifically, the fruit and vegetable, bread, sweet-tooth, and red meat patterns and MGUS. Prior studies have reported fruit and wholegrain bread consumption with lower risk of MGUS and soft drinks and red meat consumption with increased risk of MGUS [19–21, 38]. We did not find specific dietary components to be associated with overall MGUS odds and were therefore unable to replicate the findings of prior studies that have associated diet with the risk of MGUS. Our study is the largest on the topic to date and includes a very well characterized cohort with nationwide population-based screening for MGUS. However, the Icelandic population is homogenous, for example, with generally a low consumption of vegetable and fruits. Although the method of classifying the dietary patterns utilizes the available variance in the data to maximize the difference between the different dietary patterns, this may have affected the results and a direct comparison is not possible, for example, between vegetarians/vegans and meat eaters, as is possible in the EPIC-Oxford cohort for example [15]. Even so, our findings in the context of prior inconsistent findings of studies on the topic is indicative of a low causal importance of diet in the development of MGUS. However, diet might affect the risk of MGUS progression to MM, which would be consistent with prior studies on diet as a risk factor for MM [15–18]. If so, diet could be a modifiable risk factor to prevent MGUS progression and future studies should focus on the association of diet and the risk of MGUS progression.

Interestingly, high consumption of dairy products was associated with a two-fold increased risk of IgA MGUS, a relationship which was dose-dependent and robust across model designs. Although associations like this could be spurious in the setting of multiple comparisons, there is some theoretical support for this potential relationship, as IgA immunoglobulin production is closely linked related to the gastrointestinal immune system [23] and the strongest evidence for diet-cancer relationships pertains to cancer of the gastrointestinal tract [39]. We have previously observed that the portion of IgA MGUS of all MGUS cases behaves differently with age from other MGUS isotypes [3]. These results corroborate the theory that the etiology of IgA MGUS differs from the other isotypes, and suggest that diet, in particular dairy consumption, may play a role. This is of further interest since IgA MGUS has been associated with a higher risk of progression to MM [5].

This study has some strengths. Firstly, it is the largest study on the association of diet and MGUS to date and based on a screened MGUS cohort. Secondly, by assessing diet as specific components and patterns, this study can assess the complex and interdependent relationship between dietary components and comprehensively assess the association of the diet as a whole with MGUS. Finally, due to the large size of this study, it was possible to assess the association of diet and different MGUS isotypes.

The study also has important limitations. Firstly, data on the exposure, i.e. dietary habits, are collected years after MGUS diagnosis and the assumption made that this is, on average, reflective of long-term dietary habits. The reverse temporal relationship of the exposure and outcome data precludes definitive assignment of causality. However, studies have shown that data from FFQ are consistent in most individuals in the medium-term and this design is similar to that of prior studies in the field [40]. Secondly, using a FFQ risks recall bias [41]. and social desirability bias may also have affected the results. These biases affects studies that rely on self-reporting of dietary habits [42]. Reporting bias may also occur due to social pressure, but this risk is mitigated by our use of a validated FFQ [42]. Furthermore, respondents to an FFQ may be more interested in diet overall and those with a healthier diet may be more likely to be captured in the data, however this is not differential between those with and without MGUs. Although the use of PCA gives an informative overview of the dietary patterns of the population in iStopMM, its ability for direct comparison between studies and differences in diets in different populations. Thirdly, individuals that know about their MGUS diagnosis may knowingly or unknowingly change their dietary intake [43]. However, a sensitivity analysis showed no difference in dietary patterns or dietary item consumption between those who are aware and unaware of their MGUS status (Supplementary Table 9), suggesting that this bias did not affect the current study. Fourth, the Icelandic population is genetically homogenous and mainly of white ethnicity [44], possibly limiting the generalizability of the findings to other populations. Finally, Icelanders have been shown to have very low consumption of fruits, vegetables, and wholegrains in general [45] and most participants in the study had low consumption of these dietary components, which may have limited the study´s ability to detect effects of these food items.

In conclusion, this large study of over 27,200 individuals screened for MGUS indicates that dietary patterns or specific dietary components are unlikely to be strongly associated with the overall risk of developing MGUS. Interestingly, we observed a robust and dose-dependent relationship between higher dairy consumption and an increased risk of IgA MGUS, suggesting a unique relationship between dairy consumption and this small but higher risk subgroup with MGUS. The findings of this cross-sectional study suggest that dietary habits are not associated with MGUS, although a possible exception of an association of high dairy consumption and increased risk of IgA MGUS. Although dietary habits may have some effects on MGUS development the findings of this study indicate that dietary habits are not a major risk factor for the development of MGUS and, by extension, not a major causal factor in the early stages of MM development.

## Supplementary information

Supplemental material for Dietary intake and the risk of monoclonal gammopathy of undetermined significance: Results from the population-based iStopMM screening study

Food frequency questionnaire

## Data Availability

The datasets generated during and/or analyzed during the current study are not publicly available due to participant privacy, but are available from the corresponding author on reasonable request, pending the approval of the iStopMM study team and Icelandic National Bioethics Committee.

## References

[1] Landgren O, Kyle RA, Pfeiffer RM, Katzmann JA, Caporaso NE, Hayes RB, et al. Monoclonal gammopathy of undetermined significance (MGUS) consistently precedes multiple myeloma: a prospective study. Blood. 2009;113:5412–7. 19179464 10.1182/blood-2008-12-194241 PMC2689042

[2] Kyle RA, Durie BG, Rajkumar SV, Landgren O, Blade J, Merlini G, et al. Monoclonal gammopathy of undetermined significance (MGUS) and smoldering (asymptomatic) multiple myeloma: IMWG consensus perspectives risk factors for progression and guidelines for monitoring and management. Leukemia. 2010;24:1121–7. 20410922 10.1038/leu.2010.60 PMC7020664

[3] Love TJ, Rögnvaldsson S, Thorsteinsdottir S, Aspelund T, Reed ER, Vidarsson B, et al. Prevalence of MGUS is high in the istopmm study but the prevalence of IgA MGUS does not increase with age in the way other immunoglobulin subtypes do. Blood. 2022;140:256–8.

[4] Kyle RA, Larson DR, Therneau TM, Dispenzieri A, Kumar S, Cerhan JR, et al. Long-term follow-up of monoclonal gammopathy of undetermined significance. N Engl J Med. 2018;378:241–9. 29342381 10.1056/NEJMoa1709974 PMC5852672

[5] Kyle RA, Therneau TM, Rajkumar SV, Larson DR, Plevak MF, Offord JR, et al. Prevalence of monoclonal gammopathy of undetermined significance. N Engl J Med. 2006;354:1362–9. 16571879 10.1056/NEJMoa054494

[6] Castaneda-Avila MA, Ulbricht CM, Epstein MM. Risk factors for monoclonal gammopathy of undetermined significance: a systematic review. Ann Hematol. 2021;100:855–63. 33416902 10.1007/s00277-021-04400-7 PMC8349467

[7] Landgren O, Kristinsson SY, Goldin LR, Caporaso NE, Blimark C, Mellqvist UH, et al. Risk of plasma cell and lymphoproliferative disorders among 14621 first-degree relatives of 4458 patients with monoclonal gammopathy of undetermined significance in Sweden. Blood. 2009;114:791–5. 19182202 10.1182/blood-2008-12-191676 PMC2716021

[8] Wadhera RK, Rajkumar SV. Prevalence of monoclonal gammopathy of undetermined significance: a systematic review. Mayo Clin Proc. 2010;85:933–42. 20713974 10.4065/mcp.2010.0337 PMC2947966

[9] Thordardottir M, Lindqvist EK, Lund SH, Costello R, Burton D, Korde N, et al. Obesity and risk of monoclonal gammopathy of undetermined significance and progression to multiple myeloma: a population-based study. Blood Adv. 2017;1:2186–92. 29296866 10.1182/bloodadvances.2017007609 PMC5737120

[10] Landgren O, Rajkumar SV, Pfeiffer RM, Kyle RA, Katzmann JA, Dispenzieri A, et al. Obesity is associated with an increased risk of monoclonal gammopathy of undetermined significance among black and white women. Blood. 2010;116:1056–9. 20421448 10.1182/blood-2010-01-262394 PMC2938127

[11] Landgren O, Graubard BI, Katzmann JA, Kyle RA, Ahmadizadeh I, Clark R, et al. Racial disparities in the prevalence of monoclonal gammopathies: a population-based study of 12,482 persons from the National Health and Nutritional Examination Survey. Leukemia. 2014;28:1537–42. 24441287 10.1038/leu.2014.34 PMC4090286

[12] Brown LM, Gridley G, Pottern LM, Baris D, Swanso CA, Silverman DT, et al. Diet and nutrition as risk factors for multiple myeloma among blacks and whites in the United States. Cancer Causes Control. 2001;12:117–25. 11246840 10.1023/a:1008937901586

[13] Hosgood HD 3rd, Baris D, Zahm SH, Zheng T, Cross AJ. Diet and risk of multiple myeloma in Connecticut women. Cancer Causes Control. 2007;18:1065–76. 17694422 10.1007/s10552-007-9047-z

[14] Vlajinac HD, Pekmezović TD, Adanja BJ, Marinković JM, Kanazir MS, Suvajdzić ND, et al. Case-control study of multiple myeloma with special reference to diet as risk factor. Neoplasma. 2003;50:79–83. 12687283

[15] Key TJ, Appleby PN, Crowe FL, Bradbury KE, Schmidt JA, Travis RC. Cancer in British vegetarians: updated analyses of 4998 incident cancers in a cohort of 32,491 meat eaters, 8612 fish eaters, 18,298 vegetarians, and 2246 vegans. Am J Clin Nutr. 2014;100:378s–85s. 24898235 10.3945/ajcn.113.071266 PMC4144109

[16] Castro F, Parikh R, Eustaquio JC, Derkach A, Joseph JM, Lesokhin AM, et al. Pre-diagnosis dietary patterns and risk of multiple myeloma in the NIH-AARP diet and health study. Leukemia. 2024;38:438–41. 38158443 10.1038/s41375-023-02132-3 PMC10919351

[17] Lee DH, Fung TT, Tabung FK, Colditz GA, Ghobrial IM, Rosner BA, et al. Dietary pattern and risk of multiple myeloma in two large prospective US cohort studies. JNCI Cancer Spectr. 2019;3:pkz025. 31149654 10.1093/jncics/pkz025 PMC6532330

[18] Lee DH, Fung TT, Tabung FK, Marinac CR, Devore EE, Rosner BA, et al. Prediagnosis dietary pattern and survival in patients with multiple myeloma. Int J Cancer. 2020;147:1823–30. 32067221 10.1002/ijc.32928 PMC7423719

[19] Joseph JM, Hillengass J, Tang L, Lesokhin AM, Landgren O, Usmani SZ, et al. Dietary risk factors for monoclonal gammopathy of undetermined significance in a racially diverse population. Blood Adv. 2024;8:538–48. 38055924 10.1182/bloodadvances.2023011608 PMC10835229

[20] Schmidt B, Debold E, Frank M, Arendt M, Dragano N, Dürig J, et al. Socioeconomic position is positively associated with monoclonal gammopathy of undetermined significance in a population-based cohort study. Ann Hematol. 2019;98:2761–7. 31691002 10.1007/s00277-019-03825-5

[21] Thordardottir M, Lindqvist EK, Lund SH, Costello R, Burton D, Steingrimsdottir L, et al. Dietary intake is associated with risk of multiple myeloma and its precursor disease. PLoS ONE. 2018;13:e0206047. 30383820 10.1371/journal.pone.0206047 PMC6211667

[22] Owen RG, Treon SP, Al-Katib A, Fonseca R, Greipp PR, McMaster ML, et al. Clinicopathological definition of Waldenstrom’s macroglobulinemia: consensus panel recommendations from the Second International Workshop on Waldenstrom’s Macroglobulinemia. Semin Oncol. 2003;30:110–5. 12720118 10.1053/sonc.2003.50082

[23] Wang X, Hao GL, Wang BY, Gao CC, Wang YX, Li LS, et al. Function and dysfunction of plasma cells in intestine. Cell Biosci. 2019;9:26. 30911371 10.1186/s13578-019-0288-9 PMC6417281

[24] Rögnvaldsson S, Love TJ, Thorsteinsdottir S, Reed ER, Óskarsson JÞ, Pétursdóttir Í, et al. Iceland screens, treats, or prevents multiple myeloma (iStopMM): a population-based screening study for monoclonal gammopathy of undetermined significance and randomized controlled trial of follow-up strategies. Blood Cancer J. 2021;11:94. 34001889 10.1038/s41408-021-00480-w PMC8128921

[25] Andresdottir E, Birgisdottir BE, Torfadottir JE. Assessing the validity of the food frequency questionnaire used in the SAGA cohort study with a special focus on fish consumption. University of Iceland; 2023.

[26] Watkins MW. Exploratory factor analysis: a guide to best practice. J Black Psychol. 2018;44:219–46.

[27] Newby PK, Tucker KL. Empirically derived eating patterns using factor or cluster analysis: a review. Nutr Rev. 2004;62:177–203. 15212319 10.1301/nr.2004.may.177-203

[28] Kaiser HF. The varimax criterion for analytic rotation in factor analysis. Psychometrika. 1958;23:187–200.

[29] Stavseth MR, Clausen T, Røislien J. How handling missing data may impact conclusions: a comparison of six different imputation methods for categorical questionnaire data. SAGE Open Med. 2019;7:2050312118822912. 30671242 10.1177/2050312118822912 PMC6329020

[30] Buuren SV. Flexible imputation of missing data. 2nd ed. New York: Chapman and Hall/CRC; 2018.

[31] R Core Team. R: a language and environment for statistical computing. R Foundation for Statistical Computing: Vienna, Austria; 2022.

[32] van Buuren S, Groothuis-Oudshoorn K. mice: Multivariate imputation by chained equations in R. J Stat Softw. 2011;45:1–67.

[33] Lê S, Josse J, Husson F. FactoMineR: an R package for multivariate analysis. J Stat Softw. 2008;25:1–18.

[34] Kassambara A, Mundt F. factoextra: Extract and visualize the results of multivariate data analyses*_*. R package version 1.0.7; 2020.

[35] Kuhn M, Jackson S, Cimentada J. corrr: Correlations in R. 2022.

[36] Raiche G, Magis D. nFactors: Parallel Analysis and Other Non Graphical Solutions to the Cattell Scree Test. 2022.

[37] Bernaards CA, Jennrich RI. Gradient projection algorithms and software for arbitrary rotation criteria in factor analysis. Educ Psychol Meas. 2005;65:676–96.

[38] Joseph JM, Tang L, Hillengass J, Moysich K, Landgren O, Usmani S, et al. Low intake of fruits and vegetables and high intake of processed meats and juices are associated with risk of MGUS in the National Health and Nutrition Examination Survey (NHANES) Database. Blood. 2022;140:12556–8.

[39] Key TJ, Bradbury KE, Perez-Cornago A, Sinha R, Tsilidis KK, Tsugane S. Diet, nutrition, and cancer risk: what do we know and what is the way forward? BMJ. 2020;368:m511. 32139373 10.1136/bmj.m511 PMC7190379

[40] Thordardottir M, Lindqvist EK, Lund SH, Costello R, Torfadottir JE, Birgisdottir BE, et al. Dietary pattern and risk of monoclonal gammopathy of undetermined significance: a population-based study. Blood. 2016;128:3257–3257.

[41] Naska A, Lagiou A, Lagiou P. Dietary assessment methods in epidemiological research: current state of the art and future prospects. F1000Res. 2017;6:926. 28690835 10.12688/f1000research.10703.1 PMC5482335

[42] Willett W. Nutritional epidemiology. Oxford University Press; 2012.

[43] Malik MA, Sweeney NW, Jafri M, Derkach A, Chmielewski C, Adintori PA, et al. Nutrition perceptions, needs and practices among patients with plasma cell disorders. Blood Cancer J. 2022;12:70. 35443718 10.1038/s41408-022-00666-w PMC9021216

[44] Gudbjartsson DF, Helgason H, Gudjonsson SA, Zink F, Oddson A, Gylfason A, et al. Large-scale whole-genome sequencing of the Icelandic population. Nat Genet. 2015;47:435–44. 25807286 10.1038/ng.3247

[45] Gunnarsdóttir S, Guðmannsdóttir R, Þorgeirsdóttir H, Torfadóttir JE, Steingrímsdóttir L, Tryggvadóttir EA, et al. Hvað borða Íslendingar? Könnun á mataræði Íslendinga 2019–2021: Helstu niðurstöður og samanburður við könnun frá 2010–2011 [What do Icelanders eat? Icelandic Diet Survey 2019–2021: Main results and comparison with the 2010–2011 survey]. Reykjavík: Directorate of Health and Nutritional Research Unit, University of Iceland; 2022. https://maturinnokkar.hi.is/wp-content/uploads/2022/03/Hvadbordaislendingar_vefur_endanlegt.pdf.

