# Supplementary material for: Dietary intake and the risk of monoclonal gammopathy of undetermined significance: results from the population-based iStopMM screening study
Source: Blood Cancer J. 2026 Apr 8;16(1):77. doi: 10.1038/s41408-026-01480-4 (PMC13187420; doi:10.1038/s41408-026-01480-4)
Supplement: Supplementary file 1 — Supplemental material for Dietary intake and the risk of monoclonal gammopathy of undetermined significance: Results from the population-based iStopMM screening study [file 41408_2026_1480_MOESM1_ESM.docx]

**Supplement**

Dietary intake and the risk of monoclonal gammopathy of undetermined significance: Results from the population-based iStopMM screening study

# Supplemental tables

Supplemental table 1. Comparison of background characteristics of FFQ cohort vs. rest of iStopMM cohort

|  | **Answered FFQ** | **Unanswered FFQ** | **p-value** |
| --- | --- | --- | --- |
| n | 27217 | 48205 |  |
| MGUS^a^ (%) | 1020 (3.7%) | 2360 (4.9%) | <0.001 |
| Sex^a^ (%)   - Males - Females | 12260 (45%)  15011 (55%) | 21929 (45.5%)  26276 (54.5%) | 0.241 |
| Age^b^ (Q1-Q3) | 59 (52-66) | 63 (54-72) | <0.001 |

a: Pearson´s Chi-square test

b: Wilcoxon´s test

Supplemental Table 2. FFQ-answer percentage

| FFQ-answer percentage | N (mgus) |
| --- | --- |
| 100% | 15106 (494) |
| 90-99% | 10201 (434) |
| 80-89% | 628 (34) |
| 70-79% | 211 (6) |
| 60-69% | 198 (10) |
| 50-59% | 167 (10) |
| <50% | 706 (32) |

| FFQ-answer percentage | N (mgus) | % of study population  (% of total MGUS) |
| --- | --- | --- |
| 100% | 15106 (494) | 55.5% (48.4%) |
| ≥ 90% | 25307 (928) | 93.0% (91.1%) |
| ≥ 80% | 25935 (962) | 95.3% (94.3%) |
| ≥ 70% | 26146 (968) | 96.1% (94.9%) |
| ≥ 60% | 26344 (978) | 96.8% (95.9%) |
| ≥ 50% | 26511 (988) | 97.4% (96.9%) |
| <50% | 27217 (1020) | 100% |

In total, 25,307 (93% of individuals, 91% of MGUS cases) had an answer rate equal to or greater than 90% for the FFQ-variables.

Assumptions for imputation of missing values were met, showing that results for the imputed data are reliable and valid. Furthermore, the complete case analysis does not show notable differences in the results.

Supplemental table 3. Dietary Patterns extracted from Principal Component Analysis

| **Name of Pattern** | **Key component loadings^a^** | **% Variance explained^b^** |
| --- | --- | --- |
| Fruit & Vegetable Pattern | Fruits & Berries – 0.69  Nuts & Seeds – 0.68  Raw Vegetables – 0.64  Dried Fruit – 0.58  Cooked Vegetables – 0.52  Vegetarian Meals – 0.50  Vegetable & Fruit Toppings – 0.48  Oatmeal – 0.40  Hummus & Pesto – 0.37  Skyr^c^ & Yoghurt – 0.34 | 7.18% |
| Red Meat Pattern | Minced Meat – 0.70  Fatty Red Meat – 0.67  Processed Meat – 0.56  Sauce – 0.47  Meat Toppings – 0.38  Fried Potatoes – 0.37 | 7.15% |
| Sweet Tooth Pattern | Candy – 0.73  Milk Chocolate – 0.65  Chips, Popcorn & Pretzels – 0.57  Ice Cream – 0.43  Pizza – 0.32 | 5.09% |
| Bread Pattern | Cheese Toppings – 0.69  Wholegrain bread – 0.61  Crispbread – 0.60  Rye & Flatbread – 0.52  Jam & Marmalade – 0.38  Vegetable & Fruit Toppings – 0.35  Meat Toppings – 0.31 | 3.07% |
| Fish Meal Pattern | Lean Fish – 0.69  Salted Fish – 0.63  Fatty Fish – 0.56  Boiled Potatoes – 0.43 | 2.12% |

a: Component loadings for dietary products from PCA. Loadings <-0.30 and >0.30 included in table.

b: Percentage of variance explained by the dietary pattern.

c: Skyr is a traditional Icelandic cultured high-protein and low-fat dairy product.

d: Flatbread is a traditional Icelandic bread baked from whole wheat flour and rye.

Supplemental table 4. Principal component number, Eigenvalue, and percentage of variance explained

| **Dietary Pattern** | **Principal Component number** | **Eigenvalue** | **% of Variance Explained** |
| --- | --- | --- | --- |
| Vegetable & Fruit Pattern | Nr. 1 | 4.92 | 7.34% |
| Red Meat Pattern | Nr. 2 | 4.55 | 6.79% |
| Sweet Tooth Pattern | Nr. 3 | 3.40 | 5.08% |
| Bread Pattern | Nr. 4 | 2.10 | 3.14% |
| Fish Meal Pattern | Nr. 9 | 1.34 | 1.99% |

Supplemental table 5. Associations between dietary intake and MGUS - Fully adjusted (Age, sex, education, physical activity, BMI)

| **Dietary Pattern** | **0-25th percentile**  **(n = 5556)** | **25-50th percentile**  **(n = 5658)** | **50-75th percentile**  **(n=5616)** | **75th-100th percentile**  **(n=5633)** |
| --- | --- | --- | --- | --- |
| **n = 22463** | **OR (95% CI)** | **OR (95%CI)** | **OR (95%CI)** | **OR (95% CI)** |
| Fruit & Vegetable Pattern | Ref. | 1.17 (0.85-1.62) | 0.88  (0.62-1.25) | 1.23  (0.87-1.73) |
| Red Meat Pattern | Ref. | 0.76 (0.55-1.04) | 0.73  (0.53-1.01) | 0.85  (0.62-1.17) |
| Sweet Tooth Pattern | Ref. | 1.27 (0.93-1.74) | 1.26  (0.91-1.74) | 1.05  (0.73-1.49) |
| Bread Pattern | Ref. | 1.21 (0.85-1.73) | 1.38  (0.98-1.96) | 1.24  (0.88-1.76) |
| Fish Meal Pattern | Ref. | 0.98 (0.71-1.36) | 0.94  (0.68-1.30) | 0.90  (0.65-1.24) |
|  | | | | |
| **Dietary Component** | **0-25^th^ percentile** | **25^th^-50^th^ percentile** | **50^th^-75^th^ percentile** | **75^th^-100^th^ percentile** |
|  | OR (95% CI) | OR (95% CI) | OR (95% CI) | OR (95% CI) |
| Meat products | Ref. | 1.32 (0.184-2.76) | 0.27 (0.08-8.94) | 0.40 (0.10-1.56) |
| Red meat | Ref. | 1.59 (0.38-7.01) | 0.75 (0.16-4.25) | 0.41 (0.11-1.40) |
| Poultry | Ref. | 0.90 (0.31-2.62) | | 1.67 (0.44-8.13) |
| Fish | Ref. | 0.40 (0.09-1.52) | 0.42 (0.09-1.94) | 1.05 (0.22-4.82) |
| Dairy | Ref. | 0.56 (0.126-2.25) | 3.30 (0.57-2.63) | 1.12 (0.25-4.41) |
| Fruits | Ref. | 1.06 (0.89-1.26) | | 1.00 (0.86-1.15) |
| Vegetables | Ref. | 1.96 (0.54-0.06) | 4.91 (0.83-9.43) | 1.33 (0.42-4.72) |
| Wholegrain bread | Ref. | 0.80 (0.22-3.11) | | 1.00 (0.30-3.16) |

Abbreviations: OR; Odds Ratio, 95% CI; 95% Confidence interval.

Supplemental Table 6. Associations between the different dietary patterns and MGUS – Complete Case Analysis – Fully adjusted model

| **Dietary Pattern** | **Quartile 1** | **Quartile 2** | **Quartile 3** | **Quartile 4** |
| --- | --- | --- | --- | --- |
| **n = 15106** | **OR (95% CI)** | **OR (95%CI)** | **OR (95% CI)** | **OR (95% CI)** |
| Vegetable & Fruit Pattern | Ref. | 0.86 (0.66-1.11) | 0.87 (0.67-1.13) | 0.87 (0.66-1.14) |
| Red Meat Pattern | Ref. | 0.92 (0.71-1.20) | 0.98 (0.75-1.27) | 0.95 (0.773-1.24) |
| Sweet Tooth Pattern | Ref. | 1.11 (0.87-1.42) | 1.02 (0.79-1.32) | 1.04 (0.79-1.37) |
| Bread Pattern | Ref. | 1.26 (0.97-1.63) | 0.86 (0.65-1.13) | 0.811 (0.62-1.07) |
| Fish Meal Pattern | Ref. | 0.98 (0.75-1.27) | 1.13 (0.88-1.46) | 0.94 (0.72-1.21) |

Supplemental Table 7. Associations between the different dietary components and MGUS - Complete Case Analysis - Fully adjusted model

| **Dietary Component** | **Quartile 1** | **Quartile 2** | **Quartile 3** | **Quartile 4** |
| --- | --- | --- | --- | --- |
| **n = 15106** | **OR (95% CI)** | **OR (95%CI)** | **OR (95% CI)** | **OR (95% CI)** |
| Vegetables | Ref. | 0.94 (0.72-1.22) | 0.82 (0.62-1.07) | 0.93 (0.73-1.19) |
| Red meat | Ref. | 1.07 (0.84-1.35) | 1.12 (0.80-1.55) | 1.00 (0.76-1.30) |
| Fish | Ref. | 0.93 (0.70-1.24) | 1.08 (0.80-1.45) | 1.17 (0.93-1.47) |
| Dairy | Ref. | 1.16 (0.89-1.51) | 1.07 (0.81-1.41) | 1.00 (0.77-1.29) |
| Fruits* | Ref. | 1.22 (0.95-1.55) | 1.06 (0.85-1.32) | |
| Wholegrain* | Ref. | 1.07 (0.84-1.36) | 0.91 (0.73-1.13) | |

*Due to low variance in distribution of fruit consumption and wholegrain consumption individuals were grouped into tertiles instead of quartiles

In total, 15,106 participants answered all 67 questions used in the PCA. Of those, 475 participants were diagnosed with MGUS at screening (3.14%). The model adjusted for age, sex, weekly physical activity, and education level. No significant results were observed for the dietary patterns.

Supplemental Table 8. CCA of the association between dairy consumption and IgA MGUS

| **Dairy** | **0-25th percentile** | **25-50th percentile** | **50-75th percentile** | **75-100th percentile** |
| --- | --- | --- | --- | --- |
| **n IgA MGUS:**  **60** | **OR (95% CI)** | **OR (95%CI)** | **OR (95%CI)** | **OR (95% CI)** |
| **Crude** | Ref. | 1.60 (0.65-4.22) | 1.95 (0.80-5.08) | 2.63 (1.18-6.45) |
| **Adjusted for age & sex** | Ref. | 1.64 (0.66-4.29) | 2.07 (0.84-5.43) | **2.95 (1.30-7.38)*** |
| **Adjusted for age, sex, education, & physical activity** | Ref. | 1.60 (0.64-4.27) | 1.95 (0.78-5.17) | **2.95 (1.28-7.5)*** |

*P-value <0.015

The complete case analysis 15,106 participants with 60 MGUS cases showed significantly higher odds of having IgA MGUS for individuals in the highest quartile of dairy consumption.

Supplemental Table 9. Sensitivity Analysis - Including only MGUS individuals unknowing of their diagnosis

| **Dietary Pattern** | **Quartile 1** | **Quartile 2** | **Quartile 3** | **Quartile 4** |
| --- | --- | --- | --- | --- |
| **n = 26405 (301 MGUS)** | **OR (95% CI)** | **OR (95%CI)** | **OR (95% CI)** | **OR (95% CI)** |
| Vegetable & Fruit Pattern | Ref. | 1.10 (0.80-1.50) | 0.86 (0.61-1.20) | 1.10 (0.80-1.53) |
| Red Meat Pattern | Ref. | 0.90 (0.65-1.24) | 0.90 (0.65-1.24) | 0.93 (0.67-1.28) |
| Sweet Tooth Pattern | Ref. | 1.25 (0.92-1.70) | 1.17 (0.85-1.62) | 1.07 (0.75-1.52) |
| Bread Pattern | Ref. | 1.15 (0.83-1.60) | 0.92 (0.66-1.29) | 0.83 (0.60-1.18) |
| Fish Meal Pattern | Ref. | 0.91 (0.66-1.26) | 0.94 (0.68-1.30) | 0.96 (0.70-1.31) |

| **Dietary component** | **Quartile 1** | **Quartile 2** | **Quartile 3** | **Quartile 4** |
| --- | --- | --- | --- | --- |
| **n= 26405**  **(301 MGUS)** | **OR (95% CI)** | **OR (95%CI)** | **OR (95% CI)** | **OR (95% CI)** |
| Vegetables | Ref. | 0.90 (0.63-1.25) | 1.10 (0.80-1.51) | 1.00 (0.75-1.36) |
| Red Meat | Ref. | 1.00 (0.75-1.32) | 0.88 (0.55-1.34) | 1.03 (0.75-1.41) |
| Fish | Ref. | 0.89 (0.61-1.29) | 1.18 (0.81-1.70) | 1.17 (0.88-1.56) |
| Dairy | Ref. | 1.03 (0.72-1.46) | 1.18 (0.84-1.66) | 1.14 (0.82-1.60) |
| Fruits | Ref. | 1.04 (0.76-1.43) | 1.06 (0.82-1.38) | |
| Wholegrain | Ref. | 0.91 (0.66-1.25) | 0.92 (0.71-1.20) | |

*Due to low variance in distribution of fruit consumption and wholegrain consumption individuals were grouped into tertiles instead of quartiles

Supplemental Table 10. Association between daily wholegrain, vegetables and fruit consumption and MGUS

|  | **≤ Daily consumption** | **≥ Daily consumption** |
| --- | --- | --- |
|  | **OR (95%CI)** | |
| **Wholegrain** | **n: 21043 (713 MGUS)** | **n: 6174 (307 MGUS)** |
| **Adjusted for age & sex** | Ref. | 1.00 (0.87-1.16) |
| **Adjusted for age, sex, education, & physical activity** | Ref. | 1.05 (0.87-1.16) |
|  | | |
| **Vegetables** | **n: 14896 (592 MGUS)** | **n: 12321 (428 MGUS)** |
| **Adjusted for age & sex** | Ref. | 0.94 (0.83-1.07) |
| **Adjusted for age, sex, education, & physical activity** | Ref. | 0.95 (0.83-1.08) |
|  |  |  |
| **Fruits** | **n: 20589 (736 MGUS)** | **n: 6628 (284 MGUS)** |
| **Adjusted for age & sex** | Ref. | 1.02 (0.88-1.17) |
| **Adjusted for age, sex, education, & physical activity** | Ref. | 1.02 (0.88-1.18) |

Supplemental table 11. Weekly consumption frequency of lowest and highest quartiles for the dietary components

| Weekly Consumption- Dietary Components | Lowest quartile | Highest quartile |
| --- | --- | --- |
| Meat Products | ≤ 2.5 times per week | ≥ 4.5 times per week |
| Red Meat | ≤ 1 time per week | ≥ 3 times per week |
| Poultry | ≤ 0.5 times per week | ≥ 2 times per week |
| Fish | ≤ 1 time per week | ≥ 3 times per week |
| Dairy | ≤ 1.5 times per week | ≥ 10 times per week |
| Fruits | ≤ 1.5 times per week | ≥ 5.5 times per week |
| Vegetables | ≤ 3 times per week | ≥ 8.5 times per week |
| Wholegrain | ≤ 1.5 times per week | ≥ 5.5 times per week |

Supplemental Table 12. FFQ variable missing %

| Variable | % Missing answers |
| --- | --- |
|  |  |
| Chocomilk | 6.5 |
| Whitebread | 6.22 |
| Wholemilk_Dvit | 6.05 |
| Semi_skimmedmilk | 6 |
| Semi_skimmedmilk_Dvit | 5.98 |
| Skimmedmilk | 5.82 |
| Plant_milk | 5.62 |
| Tea | 5.6 |
| Wholemilk | 5.4 |
| Coffee_drinks_with_milk | 5.3 |
| Lactose_free_milk | 5.17 |
| Sushi | 5.03 |
| Soda_with_sugar | 4.96 |
| Milk_chocolate | 4.57 |
| Minced_meat | 4.44 |
| Candy | 4.43 |
| Vegetarian_meals | 4.38 |
| Sweet_potatoes_sidemeal | 4.36 |
| Dark_chocolate | 4.35 |
| Dried_fruit | 4.33 |
| Energy_drink | 4.31 |
| Sports_protein_drink | 4.21 |
| Biscuit | 4.17 |
| Salted_fish | 4.14 |
| Fruit_vegetable_juice | 4.1 |
| Potatoes_fries_fried_sidemeal | 4.1 |
| Mueslibar | 4.06 |
| Smoothie | 4.06 |
| Sauce_sidemeal | 4.06 |
| Nuts_seeds | 4.03 |
| Chips_popcorn_pretzels | 4.03 |
| Pasta_with_meat | 4.01 |
| Lean_fish | 4 |
| Fatty_fish | 3.99 |
| Fish_spread_on_bread | 3.94 |
| Fatty_red_meat | 3.92 |
| Cooked_vegetables | 3.87 |
| Milk_adolescence | 3.78 |
| Sugarfree_soda | 3.74 |
| Sweet_breakfastcereal | 3.71 |
| Pizza | 3.69 |
| Rice_sidemeal | 3.62 |
| Hummus_pesto_on_bread | 3.59 |
| Processed_meat | 3.53 |
| Icecream | 3.5 |
| Raw_vegetables | 3.49 |
| Rye_flat_bread | 3.44 |
| Egg | 3.44 |
| Breakfastcereal | 3.39 |
| Yoghurt_skyr_culturedmilk | 3.38 |
| Coffee | 3.37 |
| Soda_water | 3.23 |
| Meat_toppings_on_bread | 3.21 |
| Majonese_salad_on_bread | 3.16 |
| Cakes | 3.15 |
| Waffles_Pancakes | 3.08 |
| Wholegrain_sidemeal | 3.01 |
| Poultry | 2.99 |
| Potatoes_boiled_baked_sidemeal | 2.97 |
| Oatmeal_chiameal_muesli | 2.95 |
| Danishpastry_bun_icelandicdoughnut | 2.76 |
| Jam_marmelade_on_bread | 2.74 |
| Crispbread | 2.72 |
| Wholegrainbread | 2.69 |
| Fruit_berries | 2.64 |
| Vegetable_fruit_on_bread | 2.59 |
| Cheese_on_bread | 1.48 |

Supplemental Table 13. Background characteristics for the different quartile groups for Fruit & Vegetable pattern

| **Variable** | **Quartile 1** | **Quartile 2** | **Quartile 3** | **Quartile 4** | **p-value** |  |
| --- | --- | --- | --- | --- | --- | --- |
| **n** | 6805 | 6804 | 6804 | 6804 |  |  |
| **n MGUS** | 278 (4.1) | 245 (3.6) | 237 (3.5) | 260 (3.8) | 0.264 |  |
| **Measured BMI**  **(mean (SD))** | 30.35 (5.61) | 30.62 (5.89) | 28.46 (4.89) | 28.99 (6.44) | 0.093 |  |
| **sex = male (%)** | 4155 (61.1) | 3383 (49.7) | 2711 (39.8) | 2011 (29.6) | <0.001 |  |
| **Age (mean (SD))** | 58.87 (9.38) | 58.66 (9.43) | 58.95 (9.55) | 59.77 (9.73) | <0.001 |  |
| **Weekly physical activity (%)** |  |  |  |  | <0.001 |  |
| Never | 511 (7.5) | 235 (3.5) | 147 (2.2) | 79 (1.2) |  |  |
| <1 per week | 1303 (19.1) | 914 (13.4) | 606 (8.9) | 379 (5.6) |  |  |
| 1-2 per week | 1565 (23.0) | 1474 (21.7) | 1339 (19.7) | 951 (14.0) |  |  |
| 3-4 per week | 1717 (25.2) | 2115 (31.1) | 2288 (33.6) | 2227 (32.7) |  |  |
| 5-6 per week | 908 (13.3) | 1147 (16.9) | 1316 (19.3) | 1671 (24.6) |  |  |
| Daily | 801 (11.8) | 919 (13.5) | 1108 (16.3) | 1497 (22.0) |  |  |
| **Education (%)** |  |  |  |  | <0.001 |  |
| Dropped out | 209 (3.1) | 130 (1.9) | 97 (1.4) | 58 (0.9) |  |  |
| Primary School | 1603 (23.6) | 1216 (17.9) | 1058 (15.5) | 849 (12.5) |  |  |
| Secondary School | 1783 (26.2) | 1515 (22.3) | 1217 (17.9) | 917 (13.5) |  |  |
| Technical School | 1398 (20.5) | 1426 (21.0) | 1416 (20.8) | 1374 (20.2) |  |  |
| University degree | 1812 (26.6) | 2517 (37.0) | 3016 (44.3) | 3606 (53.0) |  |  |
| **MGUS Isotype (%)** |  |  |  |  | 0.956 |  |
| IgA | 31 (11.2) | 32 (13.1) | 33 (13.9) | 27 (10.4) |  |  |
| IgG | 169 (60.8) | 139 (56.7) | 141 (59.5) | 155 (59.6) |  |  |
| IgM | 60 (21.6) | 58 (23.7) | 50 (21.1) | 63 (24.2) |  |  |
| Multiple | 18 (6.5) | 16 (6.5) | 13 (5.5) | 15 (5.8) |  |  |

Supplemental Table 14. Background characteristics for the different quartile groups for Red meat pattern

| **Variable** | **Quartile 1** | **Quartile 2** | **Quartile 3** | **Quartile 4** | **p-value** |  |
| --- | --- | --- | --- | --- | --- | --- |
| **n** | 6805 | 6804 | 6804 | 6804 |  |  |
| **n MGUS** | 263 (3.9) | 253 (3.7) | 253 (3.7) | 251 (3.7) | 0.949 |  |
| **Measured BMI**  **(mean (SD))** | 29.38 (5.97) | 29.46 (6.65) | 29.37 (4.89) | 30.40 (5.64) | 0.678 |  |
| **sex = male (%)** | 2295 (33.7) | 2863 (42.1) | 3226 (47.4) | 3876 (57.0) | <0.001 |  |
| **Age (mean (SD))** | 59.68 (9.56) | 59.18 (9.55) | 58.81 (9.54) | 58.58 (9.44) | <0.001 |  |
| **Weekly physical activity (%)** |  |  |  |  | <0.001 |  |
| Never | 165 (2.4) | 208 (3.1) | 207 (3.0) | 392 (5.8) |  |  |
| <1 per week | 645 (9.5) | 738 (10.8) | 809 (11.9) | 1010 (14.8) |  |  |
| 1-2 per week | 1153 (16.9) | 1288 (18.9) | 1425 (20.9) | 1463 (21.5) |  |  |
| 3-4 per week | 2162 (31.8) | 2196 (32.3) | 2110 (31.0) | 1879 (27.6) |  |  |
| 5-6 per week | 1448 (21.3) | 1305 (19.2) | 1233 (18.1) | 1056 (15.5) |  |  |
| Daily | 1232 (18.1) | 1069 (15.7) | 1020 (15.0) | 1004 (14.8) |  |  |
| **Education (%)** |  |  |  |  | <0.001 |  |
| Dropped out | 100 (1.5) | 106 (1.6) | 123 (1.8) | 165 (2.4) |  |  |
| Primary School | 953 (14.0) | 1142 (16.8) | 1227 (18.0) | 1404 (20.6) |  |  |
| Secondary School | 920 (13.5) | 1250 (18.4) | 1416 (20.8) | 1846 (27.1) |  |  |
| Technical School | 1463 (21.5) | 1401 (20.6) | 1422 (20.9) | 1328 (19.5) |  |  |
| University degree | 3369 (49.5) | 2905 (42.7) | 2616 (38.4) | 2061 (30.3) |  |  |
| **MGUS Isotype (%)** |  |  |  |  | 0.252 |  |
| IgA | 33 (12.5) | 25 (9.9) | 34 (13.4) | 31 (12.4) |  |  |
| IgG | 161 (61.2) | 144 (56.9) | 155 (61.3) | 144 (57.4) |  |  |
| IgM | 59 (22.4) | 63 (24.9) | 54 (21.3) | 55 (21.9) |  |  |
| Multiple | 10 (3.8) | 21 (8.3) | 10 (4.0) | 21 (8.4) |  |  |

Supplemental Table 15. Background characteristics for the different quartile groups for Sweet tooth pattern

| **Variable** | **Quartile 1** | **Quartile 2** | **Quartile 3** | **Quartile 4** | **p-value** |  |
| --- | --- | --- | --- | --- | --- | --- |
| **n** | 6805 | 6804 | 6804 | 6804 |  |  |
| **n MGUS** | 306 (4.5) | 288 (4.2) | 231 (3.4) | 195 (2.9) | <0.001 |  |
| **Measured BMI**  **(mean (SD))** | 29.63 (5.48) | 30.50 (7.03) | 29.07 (3.82) | 29.22 (5.95) | 0.473 |  |
| **sex = male (%)** | 3368 (49.5) | 3083 (45.3) | 2980 (43.8) | 2829 (41.6) | <0.001 |  |
| **Age (mean (SD))** | 62.77 (9.44) | 59.87 (9.25) | 58.02 (9.08) | 55.60 (8.89) | <0.001 |  |
| **Weekly physical activity (%)** |  |  |  |  | <0.001 |  |
| Never | 258 (3.8) | 234 (3.4) | 213 (3.1) | 267 (3.9) |  |  |
| <1 per week | 712 (10.5) | 769 (11.3) | 816 (12.0) | 905 (13.3) |  |  |
| 1-2 per week | 1220 (17.9) | 1341 (19.7) | 1342 (19.7) | 1426 (21.0) |  |  |
| 3-4 per week | 2005 (29.5) | 2056 (30.2) | 2136 (31.4) | 2150 (31.6) |  |  |
| 5-6 per week | 1287 (18.9) | 1310 (19.3) | 1312 (19.3) | 1133 (16.7) |  |  |
| Daily | 1323 (19.4) | 1094 (16.1) | 985 (14.5) | 923 (13.6) |  |  |
| **Education (%)** |  |  |  |  | <0.001 |  |
| Dropped out | 151 (2.2) | 118 (1.7) | 116 (1.7) | 109 (1.6) |  |  |
| Primary School | 1367 (20.1) | 1258 (18.5) | 1130 (16.6) | 971 (14.3) |  |  |
| Secondary School | 1527 (22.4) | 1399 (20.6) | 1331 (19.6) | 1175 (17.3) |  |  |
| Technical School | 1459 (21.4) | 1415 (20.8) | 1368 (20.1) | 1372 (20.2) |  |  |
| University degree | 2301 (33.8) | 2614 (38.4) | 2859 (42.0) | 3177 (46.7) |  |  |
| **MGUS Isotype (%)** |  |  |  |  | 0.517 |  |
| IgA | 32 (10.5) | 37 (12.8) | 24 (10.4) | 30 (15.4) |  |  |
| IgG | 184 (60.1) | 166 (57.6) | 138 (59.7) | 116 (59.5) |  |  |
| IgM | 69 (22.5) | 73 (25.3) | 53 (22.9) | 36 (18.5) |  |  |
| Multiple | 21 (6.9) | 12 (4.2) | 16 (6.9) | 13 (6.7) |  |  |

Supplemental Table 16. Background characteristics for the different quartile groups for Bread pattern

| **Variable** | **Quartile 1** | **Quartile 2** | **Quartile 3** | **Quartile 4** | **p-value** |  |
| --- | --- | --- | --- | --- | --- | --- |
| **n** | 6805 | 6804 | 6804 | 6804 |  |  |
| **n MGUS** | 208 (3.1) | 260 (3.8) | 248 (3.6) | 304 (4.5) | <0.001 |  |
| **Measured BMI**  **(mean (SD))** | 30.16 (5.71) | 30.25 (5.53) | 29.49 (6.02) | 29.08 (5.71) | 0.601 |  |
| **sex = male (%)** | 3331 (48.9) | 3177 (46.7) | 3010 (44.2) | 2742 (40.3) | <0.001 |  |
| **Age (mean (SD))** | 55.97 (8.89) | 57.64 (9.24) | 59.76 (9.30) | 62.89 (9.26) | <0.001 |  |
| **Weekly physical activity (%)** |  |  |  |  | <0.001 |  |
| Never | 303 (4.5) | 244 (3.6) | 220 (3.2) | 205 (3.0) |  |  |
| <1 per week | 903 (13.3) | 873 (12.8) | 753 (11.1) | 673 (9.9) |  |  |
| 1-2 per week | 1420 (20.9) | 1405 (20.6) | 1348 (19.8) | 1156 (17.0) |  |  |
| 3-4 per week | 2014 (29.6) | 2094 (30.8) | 2174 (32.0) | 2065 (30.3) |  |  |
| 5-6 per week | 1181 (17.4) | 1219 (17.9) | 1274 (18.7) | 1368 (20.1) |  |  |
| Daily | 984 (14.5) | 969 (14.2) | 1035 (15.2) | 1337 (19.7) |  |  |
| **Education (%)** |  |  |  |  | <0.001 |  |
| Dropped out | 129 (1.9) | 116 (1.7) | 113 (1.7) | 136 (2.0) |  |  |
| Primary School | 1041 (15.3) | 1109 (16.3) | 1150 (16.9) | 1426 (21.0) |  |  |
| Secondary School | 1236 (18.2) | 1364 (20.0) | 1423 (20.9) | 1409 (20.7) |  |  |
| Technical School | 1334 (19.6) | 1347 (19.8) | 1431 (21.0) | 1502 (22.1) |  |  |
| University degree | 3065 (45.0) | 2868 (42.2) | 2687 (39.5) | 2331 (34.3) |  |  |
| **MGUS Isotype (%)** |  |  |  |  | 0.047 |  |
| IgA | 25 (12.0) | 31 (11.9) | 29 (11.7) | 38 (12.5) |  |  |
| IgG | 128 (61.5) | 163 (62.7) | 138 (55.6) | 175 (57.6) |  |  |
| IgM | 50 (24.0) | 44 (16.9) | 60 (24.2) | 77 (25.3) |  |  |
| Multiple | 5 (2.4) | 22 (8.5) | 21 (8.5) | 14 (4.6) |  |  |

Supplemental Table 17. Background characteristics for the different quartile groups for Fish meal pattern

| **Variable** | **Quartile 1** | **Quartile 2** | **Quartile 3** | **Quartile 4** | **p-value** |  |
| --- | --- | --- | --- | --- | --- | --- |
| **n** | 6805 | 6804 | 6804 | 6804 |  |  |
| **n MGUS** | 281 (4.1) | 230 (3.4) | 244 (3.6) | 265 (3.9) | 0.103 |  |
| **Measured BMI**  **(mean (SD))** | 29.30 (6.37) | 29.57 (5.57) | 30.54 (5.47) | 29.33 (5.59) | 0.593 |  |
| **sex = male (%)** | 2974 (43.7) | 2767 (40.7) | 2916 (42.9) | 3603 (53.0) | <0.001 |  |
| **Age (mean (SD))** | 59.59 (9.59) | 58.27 (9.26) | 58.36 (9.42) | 60.03 (9.72) | <0.001 |  |
| **Weekly physical activity (%)** |  |  |  |  | <0.001 |  |
| Never | 277 (4.1) | 250 (3.7) | 219 (3.2) | 226 (3.3) |  |  |
| <1 per week | 774 (11.4) | 787 (11.6) | 820 (12.1) | 821 (12.1) |  |  |
| 1-2 per week | 1252 (18.4) | 1373 (20.2) | 1352 (19.9) | 1352 (19.9) |  |  |
| 3-4 per week | 2010 (29.5) | 2108 (31.0) | 2133 (31.3) | 2096 (30.8) |  |  |
| 5-6 per week | 1306 (19.2) | 1218 (17.9) | 1233 (18.1) | 1285 (18.9) |  |  |
| Daily | 1186 (17.4) | 1068 (15.7) | 1047 (15.4) | 1024 (15.0) |  |  |
| **Education (%)** |  |  |  |  | <0.001 |  |
| Dropped out | 142 (2.1) | 100 (1.5) | 105 (1.5) | 147 (2.2) |  |  |
| Primary School | 1312 (19.3) | 1113 (16.4) | 1075 (15.8) | 1226 (18.0) |  |  |
| Secondary School | 1349 (19.8) | 1292 (19.0) | 1245 (18.3) | 1546 (22.7) |  |  |
| Technical School | 1397 (20.5) | 1404 (20.6) | 1432 (21.0) | 1381 (20.3) |  |  |
| University degree | 2605 (38.3) | 2895 (42.5) | 2947 (43.3) | 2504 (36.8) |  |  |
| **MGUS Isotype (%)** |  |  |  |  | 0.104 |  |
| IgA | 43 (15.3) | 18 (7.8) | 30 (12.3) | 32 (12.1) |  |  |
| IgG | 151 (53.7) | 147 (63.9) | 156 (63.9) | 150 (56.6) |  |  |
| IgM | 65 (23.1) | 51 (22.2) | 46 (18.9) | 69 (26.0) |  |  |
| Multiple | 22 (7.8) | 14 (6.1) | 12 (4.9) | 14 (5.3) |  |  |

Supplemental table 18 – ANOVA comparing dietary component and dietary pattern scores between Arm 1, Arm 2, Arm 3.

| Dietary components | | Anova p-value | |  | | Dietary pattern scores | | Anova p-value | | | | | |  |
| --- | --- | --- | --- | --- | --- | --- | --- | --- | --- | --- | --- | --- | --- | --- |
|  |  | |  | |  | |  | | | |  | |  | |
| Vegetables | | 0.34 | |  | | **Fruit & vegetable pattern** | | 0.24 | | | | | |  |
| Red meat |  | | 0.48 | |  | | **Red meat pattern** | | 0.45 | | | | | |
| Lean & fatty fish | | 0.06 | |  | | **Sweet tooth pattern** | | 0.44 | | | | | |  |
| Dairy |  | | 0.7 | |  | | **Bread pattern** | | 0.49 | | | | | |
| Milk |  | | 0.87 | |  | | **Fish meal pattern** | | 0.49 | | | | | |
| Fruits |  | | 0.4 | |  | |  | | | |  | |  | |
| Wholegrain | | 0.23 | |  | |  | | | |  | |  | |  |


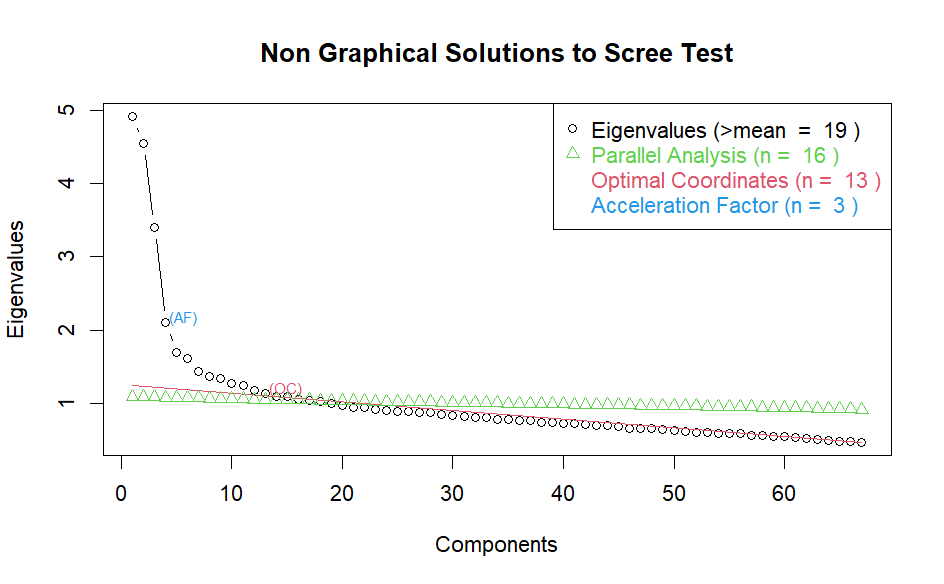


Supplemental figure 1. Parallel analysis plot


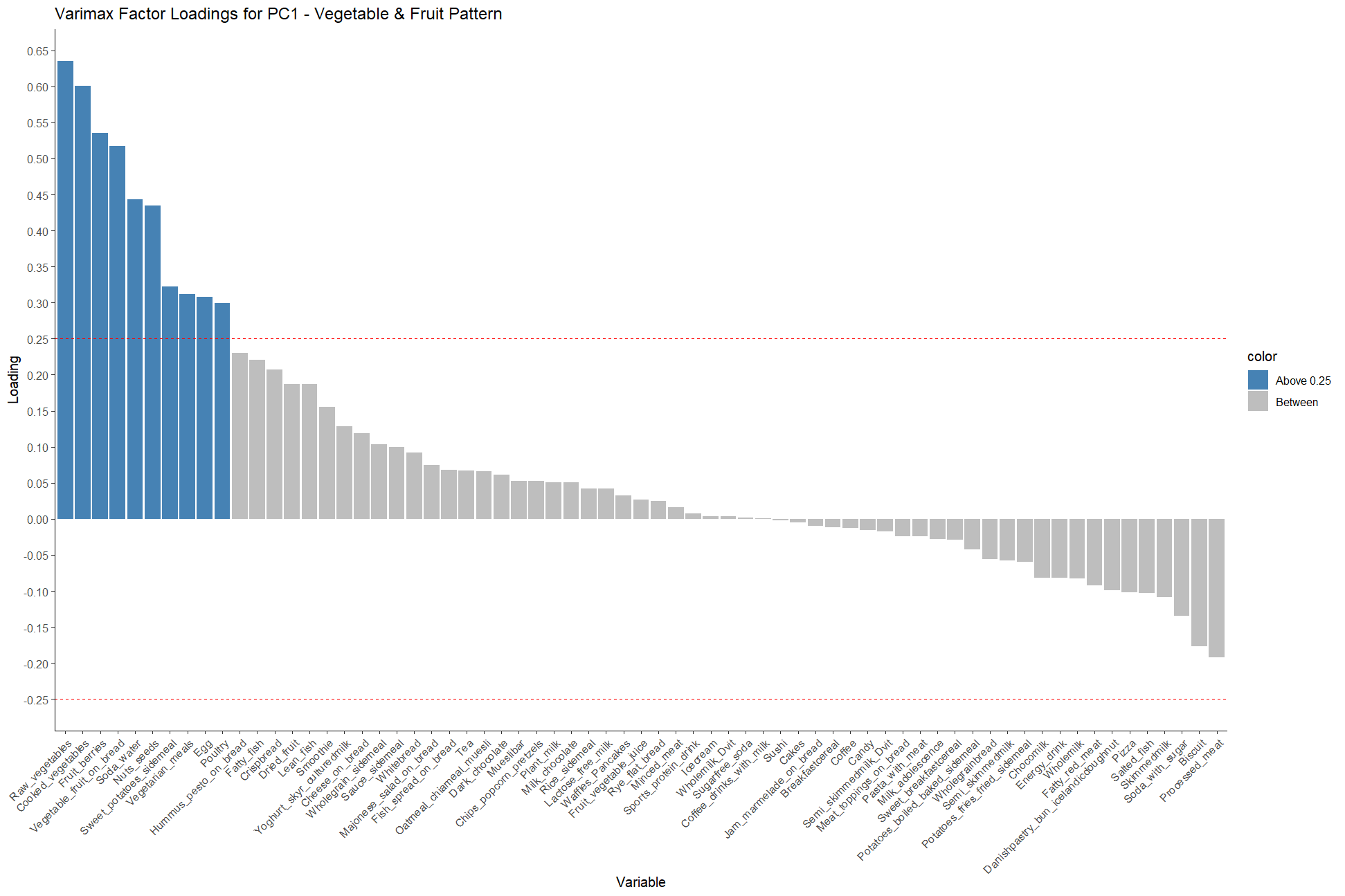


Supplemental figure 2. Vegetable & Fruit Pattern - Component Loadings Plot


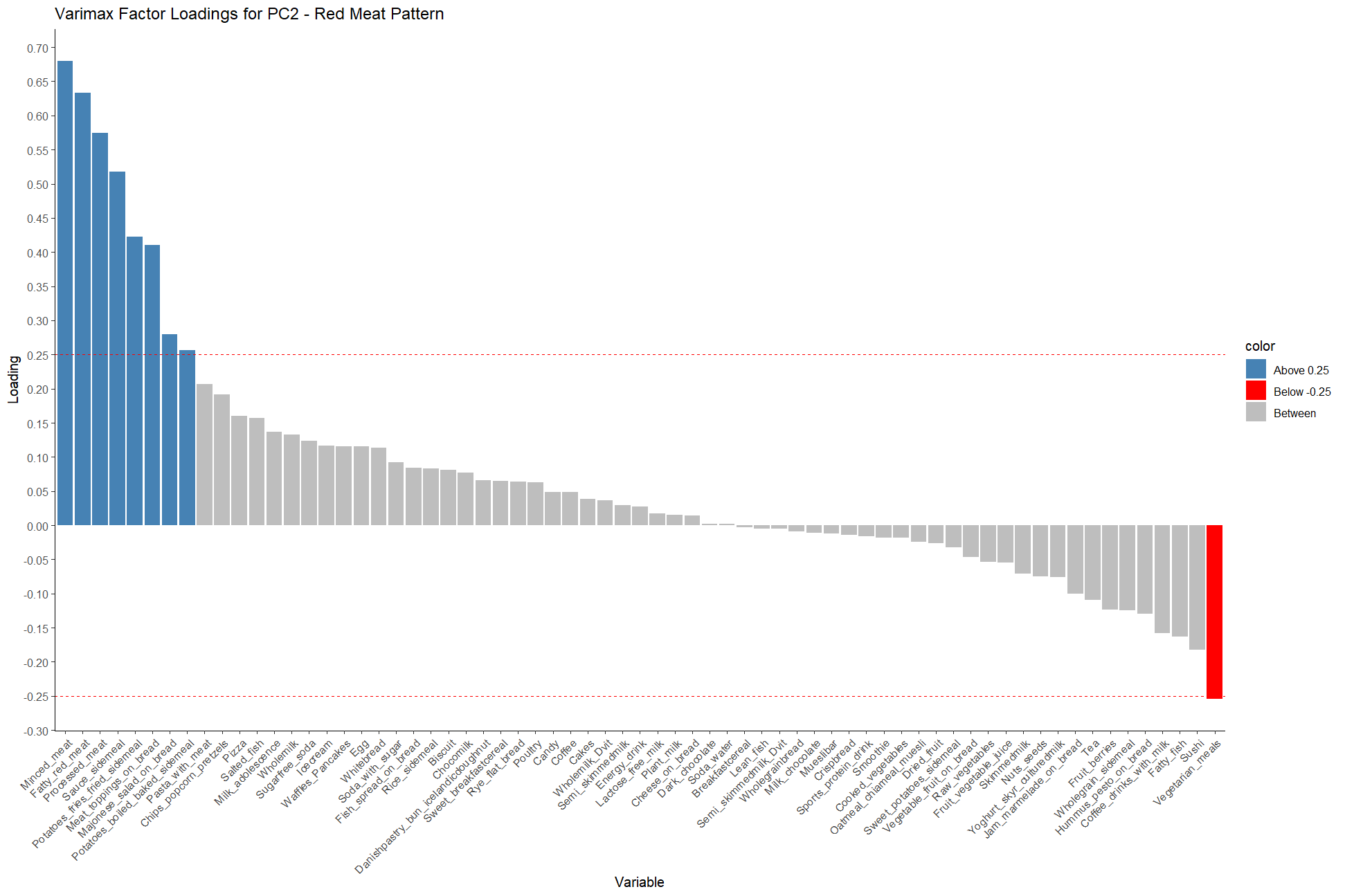


Supplemental figure 3. Red Meat Pattern - Component Loadings Plot


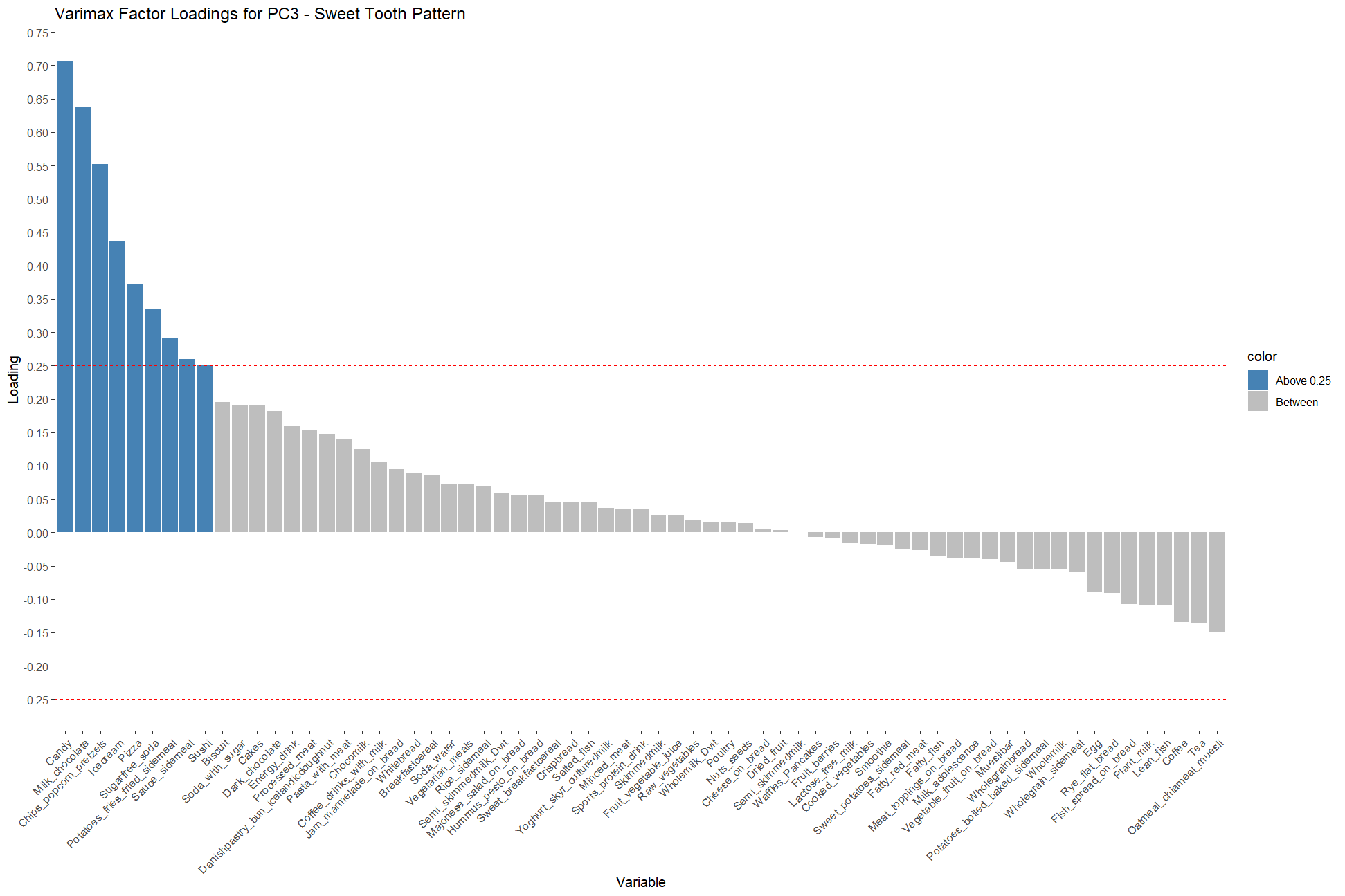


Supplemental figure 4. Sweet Tooth Pattern - Component Loadings Plot


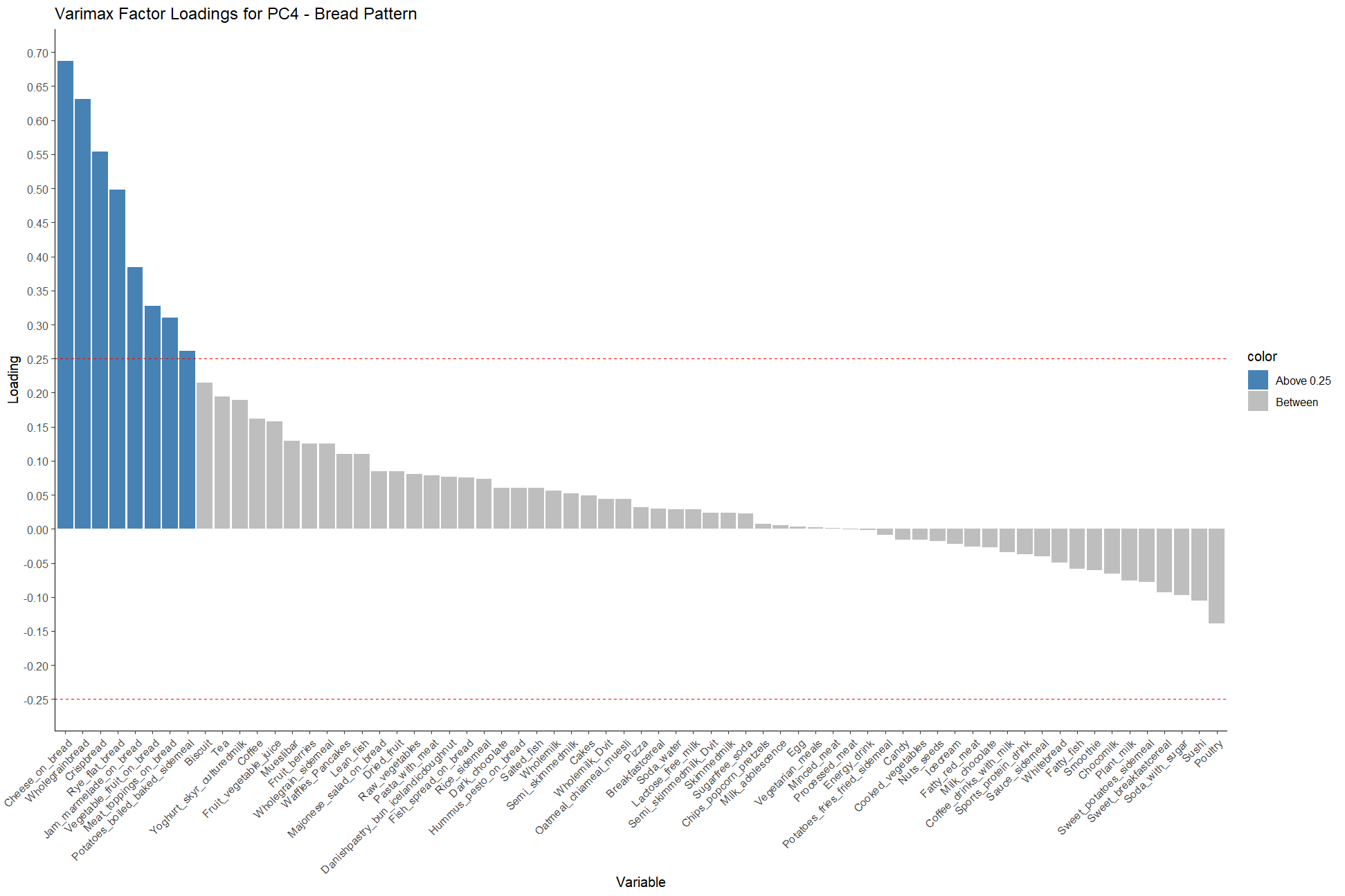


Supplemental figure 5. Bread Pattern - Component Loadings Plot


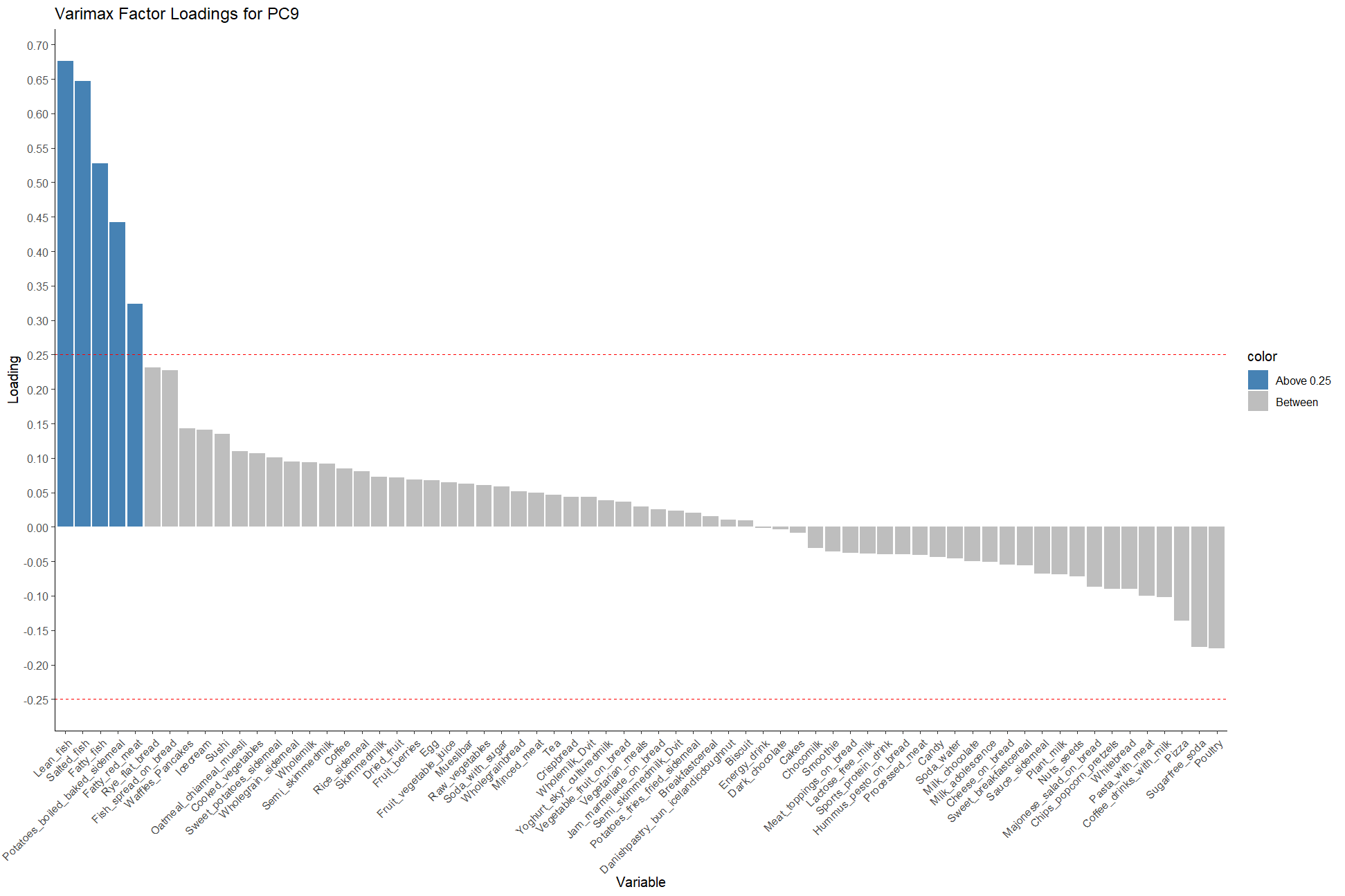


Supplemental figure 6. Fish Meal Pattern – Component loadings plot
