# Supplementary material for: Dietary intake and the risk of monoclonal gammopathy of undetermined significance: results from the population-based iStopMM screening study
Source: Blood Cancer J. 2026 Apr 8;16(1):77. doi: 10.1038/s41408-026-01480-4 (PMC13187420; doi:10.1038/s41408-026-01480-4)
Supplement: Supplementary file 2 — Food frequency questionnaire [file 41408_2026_1480_MOESM2_ESM.pdf]

Food frequency questionnaire – iStopMM

English translation of the FFQ used in *"Dietary intake and the risk of monoclonal gammopathy of undetermined significance: Results from the population-based iStopMM screening study"*

Category	***Question***	***Answering alternatives***
Meal Habits		
"How often do you eat the following?"	***Breakfast***	***1; Seldom/never<br>2; 1-3 times a month<br>3; 1-2 times a week<br>4; 3-4 times a week<br>5; 5-6 times a week<br>6: Daily/almost daily***
"How often do you eat the following?"	***Snack before lunch***	***1; Seldom/never<br>2; 1-3 times a month<br>3; 1-2 times a week<br>4; 3-4 times a week<br>5; 5-6 times a week<br>6: Daily/almost daily***
"How often do you eat the following?"	***Lunch***	***1; Seldom/never<br>2; 1-3 times a month<br>3; 1-2 times a week<br>4; 3-4 times a week<br>5; 5-6 times a***

		week<br>*6: Daily/almost daily*
"How often do you eat the following?"	*Snack after lunch*	*1; Seldom/never*<br>*2; 1-3 times a month*<br>*3; 1-2 times a week*<br>*4; 3-4 times a week*<br>*5; 5-6 times a week*<br>*6: Daily/almost daily*
"How often do you eat the following?"	*Dinner*	*1; Seldom/never*<br>*2; 1-3 times a month*<br>*3; 1-2 times a week*<br>*4; 3-4 times a week*<br>*5; 5-6 times a week*<br>*6: Daily/almost daily*
"How often do you eat the following?"	*Snack after lunch*	*1; Seldom/never*<br>*2; 1-3 times a month*<br>*3; 1-2 times a week*<br>*4; 3-4 times a week*<br>*5; 5-6 times a week*<br>*6: Daily/almost daily*
Bread products		

"How often do you eat the following types of bread/cakes/crackers/cereals?"	*White bread*	*1; Seldom/never<br>2; 1-3 times a month<br>3; 1-2 times a week<br>4; 3-4 times a week<br>5; 5-6 times a week<br>6: Daily/almost daily*
"How often do you eat the following types of bread/cakes/crackers/cereals?"	*Whole-grain bread*	*1; Seldom/never<br>2; 1-3 times a month<br>3; 1-2 times a week<br>4; 3-4 times a week<br>5; 5-6 times a week<br>6: Daily/almost daily*
"How often do you eat the following types of bread/cakes/crackers/cereals?"	*Ryebread/Flatbread*	*1; Seldom/never<br>2; 1-3 times a month<br>3; 1-2 times a week<br>4; 3-4 times a week<br>5; 5-6 times a week<br>6: Daily/almost daily*
"How often do you eat the following types of bread/cakes/crackers/cereals?"	*Crispbread/Unsweetened crackers*	*1; Seldom/never<br>2; 1-3 times a month<br>3; 1-2 times a week<br>4; 3-4 times a week<br>5; 5-6 times a week<br>6: Daily/almost daily*

"How often do you eat the following types of bread/cakes/crackers/cereals?"	*Sweet crackers*	*1; Seldom/never*<br>*2; 1-3 times a month*<br>*3; 1-2 times a week*<br>*4; 3-4 times a week*<br>*5; 5-6 times a week*<br>*6: Daily/almost daily*
"How often do you eat the following types of bread/cakes/crackers/cereals?"	*Waffles/Pancakes*	*1; Seldom/never*<br>*2; 1-3 times a month*<br>*3; 1-2 times a week*<br>*4; 3-4 times a week*<br>*5; 5-6 times a week*<br>*6: Daily/almost daily*
"How often do you eat the following types of bread/cakes/crackers/cereals?"	*Pastry*	*1; Seldom/never*<br>*2; 1-3 times a month*<br>*3; 1-2 times a week*<br>*4; 3-4 times a week*<br>*5; 5-6 times a week*<br>*6: Daily/almost daily*
"How often do you eat the following types of bread/cakes/crackers/cereals?"	*Cakes (chocolate cakes, cream cakes etc.)*	*1; Seldom/never*<br>*2; 1-3 times a month*<br>*3; 1-2 times a week*<br>*4; 3-4 times a week*<br>*5; 5-6 times a week*<br>*6: Daily/almost daily*

"How often do you eat the following types of bread/cakes/crackers/cereals?"	*Muesli bars*	*1; Seldom/never*<br>*2; 1-3 times a month*<br>*3; 1-2 times a week*<br>*4; 3-4 times a week*<br>*5; 5-6 times a week*<br>*6: Daily/almost daily*
"How often do you eat the following types of bread/cakes/crackers/cereals?"	*Oatmeal/Chia/Muesli*	*1; Seldom/never*<br>*2; 1-3 times a month*<br>*3; 1-2 times a week*<br>*4; 3-4 times a week*<br>*5; 5-6 times a week*<br>*6: Daily/almost daily*
"How often do you eat the following types of bread/cakes/crackers/cereals?"	*Breakfast cereal*	*1; Seldom/never*<br>*2; 1-3 times a month*<br>*3; 1-2 times a week*<br>*4; 3-4 times a week*<br>*5; 5-6 times a week*<br>*6: Daily/almost daily*
	Sweet breakfast cereal	*1; Seldom/never*<br>*2; 1-3 times a month*<br>*3; 1-2 times a week*<br>*4; 3-4 times a week*<br>*5; 5-6 times a week*<br>*6: Daily/almost daily*

What type of spread do you use on bread/crackers?	*Butter*<br>*Low-fat butter*<br>*Other*<br>*I don't use spread on bread/crackers*	
Bread toppings		
"How often do you eat the following toppings?"	*Cheese*	*1; Seldom/never*<br>*2; 1-3 times a month*<br>*3; 1-2 times a week*<br>*4; 3-4 times a week*<br>*5; 5-6 times a week*<br>*6: Daily/almost daily*
"How often do you eat the following toppings?"	*Meat toppings*	*1; Seldom/never*<br>*2; 1-3 times a month*<br>*3; 1-2 times a week*<br>*4; 3-4 times a week*<br>*5; 5-6 times a week*<br>*6: Daily/almost daily*
"How often do you eat the following toppings?"	*Mayonnaise salad*	*1; Seldom/never*<br>*2; 1-3 times a month*<br>*3; 1-2 times a week*<br>*4; 3-4 times a week*<br>*5; 5-6 times a week*<br>*6: Daily/almost daily*

"How often do you eat the following toppings?"	*Fish toppings*	*1; Seldom/never<br>2; 1-3 times a month<br>3; 1-2 times a week<br>4; 3-4 times a week<br>5; 5-6 times a week<br>6: Daily/almost daily*
"How often do you eat the following toppings?"	*Vegetables/Fruits as a topping*	*1; Seldom/never<br>2; 1-3 times a month<br>3; 1-2 times a week<br>4; 3-4 times a week<br>5; 5-6 times a week<br>6: Daily/almost daily*
"How often do you eat the following toppings?"	*Jam/Marmelade*	*1; Seldom/never<br>2; 1-3 times a month<br>3; 1-2 times a week<br>4; 3-4 times a week<br>5; 5-6 times a week<br>6: Daily/almost daily*
"How often do you eat the following toppings?"	*Hummus/Pesto*	*1; Seldom/never<br>2; 1-3 times a month<br>3; 1-2 times a week<br>4; 3-4 times a week<br>5; 5-6 times a week<br>6: Daily/almost daily*

Dairy products		
How often do you eat or drink the following dairy products?<br>*(Here we refer to general consumption, in cereals but not milk in coffee)*	*Whole-fat milk*	*1; Seldom/never*<br>*2; 1-3 times a month*<br>*3; 1-2 times a week*<br>*4; 3-4 times a week*<br>*5; 5-6 times a week*<br>*6: Daily/almost daily*
How often do you eat or drink the following dairy products?<br>*(Here we refer to general consumption, in cereals but not milk in coffee)*	*Skimmed milk*	*1; Seldom/never*<br>*2; 1-3 times a month*<br>*3; 1-2 times a week*<br>*4; 3-4 times a week*<br>*5; 5-6 times a week*<br>*6: Daily/almost daily*
How often do you eat or drink the following dairy products?<br>*(Here we refer to general consumption, in cereals but not milk in coffee)*	*Whole-fat milk fortified with Vitamin D*	*1; Seldom/never*<br>*2; 1-3 times a month*<br>*3; 1-2 times a week*<br>*4; 3-4 times a week*<br>*5; 5-6 times a week*<br>*6: Daily/almost daily*
How often do you eat or drink the following dairy products?<br>*(Here we refer to general consumption, in cereals but not milk in coffee)*	*Skimmed milk fortified with Vitamin D*	*1; Seldom/never*<br>*2; 1-3 times a month*<br>*3; 1-2 times a week*<br>*4; 3-4 times a week*<br>*5; 5-6 times a week*

		6: Daily/almost daily
How often do you eat or drink the following dairy products?<br>*(Here we refer to general consumption, in cereals but not milk in coffee)*	*Wholly skimmed milk*	*1; Seldom/never*<br>*2; 1-3 times a month*<br>*3; 1-2 times a week*<br>*4; 3-4 times a week*<br>*5; 5-6 times a week*<br>*6: Daily/almost daily*
How often do you eat or drink the following dairy products?<br>*(Here we refer to general consumption, in cereals but not milk in coffee)*	*Plant-milk (non-dairy milk)*	*1; Seldom/never*<br>*2; 1-3 times a month*<br>*3; 1-2 times a week*<br>*4; 3-4 times a week*<br>*5; 5-6 times a week*<br>*6: Daily/almost daily*
How often do you eat or drink the following dairy products?<br>*(Here we refer to general consumption, in cereals but not milk in coffee)*	*Lactose-free milk*	*1; Seldom/never*<br>*2; 1-3 times a month*<br>*3; 1-2 times a week*<br>*4; 3-4 times a week*<br>*5; 5-6 times a week*<br>*6: Daily/almost daily*

How often do you eat or drink the following dairy products?<br>(Here we refer to general consumption, in cereals but not milk in coffee)	*Chocolate milk*	*1; Seldom/never<br>2; 1-3 times a month<br>3; 1-2 times a week<br>4; 3-4 times a week<br>5; 5-6 times a week<br>6: Daily/almost daily*
How often do you eat or drink the following dairy products?<br>(Here we refer to general consumption, in cereals but not milk in coffee)	*Yoghurt/Sourmilk/Skyr*	*1; Seldom/never<br>2; 1-3 times a month<br>3; 1-2 times a week<br>4; 3-4 times a week<br>5; 5-6 times a week<br>6: Daily/almost daily*
"How many doses of milk or dairy products do you drink/eat per day?"		*Number submitted*
"How many doses of milk or dairy products do you drink/eat per day during adolescence?<br>(Include milk used in cereal)"		*Number submitted*
Drinks		
"How often do you drink the following drink?"		
"How often do you drink the following drink?"	*Fruit-smoothie/boozt*	*1; Seldom/never<br>2; 1-3 times a month<br>3; 1-2 times a week<br>4; 3-4 times a week<br>5; 5-6 times a week*

		6: Daily/almost daily
"How often do you drink the following drink?"	***Fruit-juice/Vegetable-juice***	***1; Seldom/never<br>2; 1-3 times a month<br>3; 1-2 times a week<br>4; 3-4 times a week<br>5; 5-6 times a week<br>6: Daily/almost daily***
"How often do you drink the following drink?"	***Sports drink or protein drink***	***1; Seldom/never<br>2; 1-3 times a month<br>3; 1-2 times a week<br>4; 3-4 times a week<br>5; 5-6 times a week<br>6: Daily/almost daily***
"How often do you drink the following drink?"	***Energy drink***	***1; Seldom/never<br>2; 1-3 times a month<br>3; 1-2 times a week<br>4; 3-4 times a week<br>5; 5-6 times a week<br>6: Daily/almost daily***

"How often do you drink the following drink?"	*Sugarfree soft drinks*	*1; Seldom/never*<br>*2; 1-3 times a month*<br>*3; 1-2 times a week*<br>*4; 3-4 times a week*<br>*5; 5-6 times a week*<br>*6: Daily/almost daily*
"How often do you drink the following drink?"	*Sugary soft drinks*	*1; Seldom/never*<br>*2; 1-3 times a month*<br>*3; 1-2 times a week*<br>*4; 3-4 times a week*<br>*5; 5-6 times a week*<br>*6: Daily/almost daily*
"How often do you drink the following drink?"	*Soda water*	*1; Seldom/never*<br>*2; 1-3 times a month*<br>*3; 1-2 times a week*<br>*4; 3-4 times a week*<br>*5; 5-6 times a week*<br>*6: Daily/almost daily*
"How often do you drink the following drink?"	*Coffee*	*1; Seldom/never*<br>*2; 1-3 times a month*<br>*3; 1-2 times a week*<br>*4; 3-4 times a week*<br>*5; 5-6 times a week*<br>*6: Daily/almost daily*

"How often do you drink the following drink?"	*Coffee drinks (Latte, Cappuccino or other coffee drinks with milk)*	*1; Seldom/never<br>2; 1-3 times a month<br>3; 1-2 times a week<br>4; 3-4 times a week<br>5; 5-6 times a week<br>6: Daily/almost daily*
"How often do you drink the following drink?"	*Tea*	*1; Seldom/never<br>2; 1-3 times a month<br>3; 1-2 times a week<br>4; 3-4 times a week<br>5; 5-6 times a week<br>6: Daily/almost daily*
"On the days that you drink sugarfree soft drinks, how much do you drink that day?"		*1; 0-250 mL<br>2; 250-500 mL<br>3; 500-750 mL<br>4; 750-1000 mL<br>5; 1000 mL or more*
On the days that you drink sugary soft drinks, how much do you drink that day?		*1; 0-250 mL<br>2; 250-500 mL<br>3; 500-750 mL<br>4; 750-1000 mL<br>5; 1000 mL or more*
"On the day that you drink coffee, how much do you drink that day?"		*1; 1 cup<br>2; 2 cups<br>3; 3 cups<br>4; 4 cups<br>5; 5 cups<br>6; 6 or more cups*
Fruits/Vegetables/Nuts		

"How often do you eat fruits, vegetables and nuts?"	*Fruits or berries*	*1; Seldom/never<br>2; 1-3 times a month<br>3; 1-2 times a week<br>4; 3-4 times a week<br>5; 5-6 times a week<br>6: Daily/almost daily*
"How often do you eat fruits, vegetables and nuts?"	*Nuts or seeds*	*1; Seldom/never<br>2; 1-3 times a month<br>3; 1-2 times a week<br>4; 3-4 times a week<br>5; 5-6 times a week<br>6: Daily/almost daily*
"How often do you eat fruits, vegetables and nuts?"	*Dried fruits*	*1; Seldom/never<br>2; 1-3 times a month<br>3; 1-2 times a week<br>4; 3-4 times a week<br>5; 5-6 times a week<br>6: Daily/almost daily*
"How often do you eat fruits, vegetables and nuts?"	*Raw vegetables*	*1; Seldom/never<br>2; 1-3 times a month<br>3; 1-2 times a week<br>4; 3-4 times a week<br>5; 5-6 times a week<br>6: Daily/almost daily*

"How often do you eat fruits, vegetables and nuts?"	*Cooked vegetables (not including potatoes)*	*1; Seldom/never<br>2; 1-3 times a month<br>3; 1-2 times a week<br>4; 3-4 times a week<br>5; 5-6 times a week<br>6: Daily/almost daily*
"On the day that you eat vegetables, how many doses of vegetables do you eat?"		*1; 1-2 doses<br>2; 3-4 doses<br>3; 5-6 doses<br>4; 7 doses or more*
Main dishes		
"How often do you eat these food products or meals as a main dish?"		
"How often do you eat these food products or meals as a main dish?"	*Poultry*	*1; Seldom never<br>2; 1-3 times a month<br>3; 1 times a week<br>4; 2 times a week<br>5; 3-4 times a week<br>6; 5-6 times a week<br>7; Daily/almost daily*
"How often do you eat these food products or meals as a main dish?"	*Meat and meat dishes (other than poultry)*	*1; Seldom never<br>2; 1-3 times a month<br>3; 1 times a week<br>4; 2 times a week<br>5; 3-4 times a week<br>6; 5-6 times a week<br>7; Daily/almost daily*

"How often do you eat these food products or meals as a main dish?"	*Lamb leg, goulash, minced meat*	*1; Seldom never*<br>*2; 1-3 times a month*<br>*3; 1 times a week*<br>*4; 2 times a week*<br>*5; 3-4 times a week*<br>*6; 5-6 times a week*<br>*7; Daily/almost daily*
"How often do you eat these food products or meals as a main dish?"	*Fat /semi-fat meat (chops, soup meat, rib steak)*	*1; Seldom never*<br>*2; 1-3 times a month*<br>*3; 1 times a week*<br>*4; 2 times a week*<br>*5; 3-4 times a week*<br>*6; 5-6 times a week*<br>*7; Daily/almost daily*
"How often do you eat these food products or meals as a main dish?"	*Processed meat (f.ex hot dogs, meat balls, bacon, sausage, nuggets)*	*1; Seldom never*<br>*2; 1-3 times a month*<br>*3; 1 times a week*<br>*4; 2 times a week*<br>*5; 3-4 times a week*<br>*6; 5-6 times a week*<br>*7; Daily/almost daily*
"How often do you eat these food products or meals as a main dish?"	*Pasta/Spaghetti dishes with meat*	*1; Seldom never*<br>*2; 1-3 times a month*<br>*3; 1 times a week*<br>*4; 2 times a week*<br>*5; 3-4 times a week*<br>*6; 5-6 times a week*<br>*7; Daily/almost daily*

"How often do you eat these food products or meals as a main dish?"	*Fish and fish meals*	*1; Seldom never*<br>*2; 1-3 times a month*<br>*3; 1 times a week*<br>*4; 2 times a week*<br>*5; 3-4 times a week*<br>*6; 5-6 times a week*<br>*7; Daily/almost daily*
"How often do you eat these food products or meals as a main dish?"	*Lean fish (cod, haddock)*	*1; Seldom never*<br>*2; 1-3 times a month*<br>*3; 1 times a week*<br>*4; 2 times a week*<br>*5; 3-4 times a week*<br>*6; 5-6 times a week*<br>*7; Daily/almost daily*
"How often do you eat these food products or meals as a main dish?"	*Fat fish (salmon, trout, atlantic wolffish, halibut)*	*1; Seldom never*<br>*2; 1-3 times a month*<br>*3; 1 times a week*<br>*4; 2 times a week*<br>*5; 3-4 times a week*<br>*6; 5-6 times a week*<br>*7; Daily/almost daily*
"How often do you eat these food products or meals as a main dish?"	*Saltfish*	*1; Seldom never*<br>*2; 1-3 times a month*<br>*3; 1 times a week*<br>*4; 2 times a week*<br>*5; 3-4 times a week*<br>*6; 5-6 times a week*<br>*7; Daily/almost daily*

"How often do you eat these food products or meals as a main dish?"	*Sushi*	*1; Seldom never<br>2; 1-3 times a month<br>3; 1 times a week<br>4; 2 times a week<br>5; 3-4 times a week<br>6; 5-6 times a week<br>7; Daily/almost daily*
"How often do you eat these food products or meals as a main dish?"	*Vegetarian dishes*	*1; Seldom never<br>2; 1-3 times a month<br>3; 1 times a week<br>4; 2 times a week<br>5; 3-4 times a week<br>6; 5-6 times a week<br>7; Daily/almost daily*
"How often do you eat these food products or meals as a main dish?"	*Pizza*	*1; Seldom never<br>2; 1-3 times a month<br>3; 1 times a week<br>4; 2 times a week<br>5; 3-4 times a week<br>6; 5-6 times a week<br>7; Daily/almost daily*
Side dishes		
"How often do you eat these food producst as a side dish?"		
"How often do you eat these food producst as a side dish?"	*Potatoes (boiled/baked)*	*1; Seldom/never<br>2; 1-3 times a month<br>3; 1-2 times a week<br>4; 3-4 times a*

		week<br>*5; 5-6 times a week*<br>*6: Daily/almost daily*
"How often do you eat these food producst as a side dish?"	*Potatoes (fried, french fries)*	*1; Seldom/never*<br>*2; 1-3 times a month*<br>*3; 1-2 times a week*<br>*4; 3-4 times a week*<br>*5; 5-6 times a week*<br>*6: Daily/almost daily*
"How often do you eat these food producst as a side dish?"	*Rice*	*1; Seldom/never*<br>*2; 1-3 times a month*<br>*3; 1-2 times a week*<br>*4; 3-4 times a week*<br>*5; 5-6 times a week*<br>*6: Daily/almost daily*
"How often do you eat these food producst as a side dish?"	*Sweet potatoes*	*1; Seldom/never*<br>*2; 1-3 times a month*<br>*3; 1-2 times a week*<br>*4; 3-4 times a week*<br>*5; 5-6 times a week*<br>*6: Daily/almost daily*

"How often do you eat these food producst as a side dish?"	*Sauces/dressings (ex. Hamburger dressing, pita dressing, brown sauce, béarnaise sauce)*	*1; Seldom/never<br>2; 1-3 times a month<br>3; 1-2 times a week<br>4; 3-4 times a week<br>5; 5-6 times a week<br>6; Daily/almost daily*
"Do you choose wholegrain or whole products as a side dish?"		*1; Never<br>2; Sometimes<br>3; Often<br>4; Always*
"How often do you eat eggs (ex. Eggs on its own, as a spread, omelet, scrambled eggs)?"		*1; Seldom/never<br>2; 1-3 times a month<br>3; 1-2 times a week<br>4; 3-4 times a week<br>5; 5-6 times a week<br>6: Daily/almost daily*
Sweets		
"How often do you eat sweets, snacks and ice cream?"	*Light chocolate*	*1; Seldom/never<br>2; 1-3 times a month<br>3; 1-2 times a week<br>4; 3-4 times a week<br>5; 5-6 times a week<br>6: Daily/almost daily*

"How often do you eat sweets, snacks and ice cream?"	*Dark chocolate*	*1; Seldom/never*<br>*2; 1-3 times a month*<br>*3; 1-2 times a week*<br>*4; 3-4 times a week*<br>*5; 5-6 times a week*<br>*6: Daily/almost daily*
"How often do you eat sweets, snacks and ice cream?"	*Other sweets than chocolate*	*1; Seldom/never*<br>*2; 1-3 times a month*<br>*3; 1-2 times a week*<br>*4; 3-4 times a week*<br>*5; 5-6 times a week*<br>*6: Daily/almost daily*
"How often do you eat sweets, snacks and ice cream?"	*Chips, tortilla chips, pop corn, pretzels*	*1; Seldom/never*<br>*2; 1-3 times a month*<br>*3; 1-2 times a week*<br>*4; 3-4 times a week*<br>*5; 5-6 times a week*<br>*6: Daily/almost daily*
"How often do you eat sweets, snacks and ice cream?"	*Ice cream, popsicles, milkshakes etc.*	*1; Seldom/never*<br>*2; 1-3 times a month*<br>*3; 1-2 times a week*<br>*4; 3-4 times a week*<br>*5; 5-6 times a week*<br>*6: Daily/almost daily*
